# Comprehensive Risk Factor Profiles, Coronary Heart Disease, and Life Expectancy in Cancer Survivors

**DOI:** 10.1016/j.jacadv.2026.102968

**Published:** 2026-07-17

**Authors:** Yang-Wei Cai, Chuan-Rui Zeng, Mao-Xiong Wu, Yi Zhang, Pin-Ming Liu, Jing-Feng Wang, Jing-Wei Gao, Hai-Feng Zhang, Yang-Xin Chen

**Affiliations:** a Department of Cardiology, Sichuan Provincial People's Hospital, School of Medicine, University of Electronic Science and Technology of China, Chengdu, China; b Department of Cardiology, Sun Yat-sen Memorial Hospital, Sun Yat-sen University, Guangzhou, China; c Guangdong Province Key Laboratory of Arrhythmia and Electrophysiology, Sun Yat-sen Memorial Hospital, Sun Yat-sen University, Guangzhou, China

**Keywords:** cancer survivor, cardio-oncology, coronary heart disease, myocardial infarction, risk factor

## Abstract

**Background:**

Cancer survivors have nearly twice the risk of coronary heart disease (CHD) compared with the general population.

**Objectives:**

We examined whether joint risk factor control was associated with cancer-related excess CHD risk and residual life expectancy.

**Methods:**

We analyzed 14,557 cancer survivors and 58,228 matched noncancer controls from the UK Biobank. Risk factor control was defined across 9 domains: blood pressure, body mass index, low-density lipoprotein cholesterol, hemoglobin A1c, renal function, smoking, physical activity, diet, and sleep. Cox proportional hazards models estimated associations with CHD, myocardial infarction (MI), and cardiovascular disease (CVD) mortality; flexible parametric survival models assessed life expectancy.

**Results:**

Over a median follow-up of 12.60 years, 1,298 CHD events, 348 MI events, and 211 CVD-related deaths occurred among cancer survivors. After adjustment for confounders, each additional controlled risk factor was associated with lower risks of CHD (HR: 0.85; 95% CI: 0.81-0.88), MI (HR: 0.81; 95% CI: 0.74-0.88), and CVD-related mortality (HR: 0.75; 95% CI: 0.67-0.84). Participants with ≥6 controlled risk factors had the lowest risks across cardiovascular outcomes. A higher degree of risk factor control was also associated with longer residual life expectancy at age 50 (33.83 vs 30.47 years in the ≥6 vs ≤ 3 controlled risk factor groups).

**Conclusions:**

The joint risk factor control is associated with a lower risk of CHD, MI, and CVD-related mortality among cancer survivors. Comprehensive assessment and optimization of modifiable risk factors may be clinically relevant for cardiovascular risk management in cardio-oncology survivorship care.

The global cancer burden continues to intensify due to population growth and aging, with projections indicating 28.4 million new cases by 2040, representing a 47% increase from 2020.^1^ Concurrently, remarkable advances in early detection and treatment have yielded substantial improvements in cancer survival, resulting in over 18 million cancer survivors in the United States as of 2022.^2^ In this evolving landscape, cardiovascular diseases (CVDs) have emerged as major life-limiting comorbidities among cancer survivors, reshaping the paradigm of survivorship care.^3^ Among cardiovascular conditions, coronary heart disease (CHD) represents a major concern because it is strongly associated with cardiovascular death and frequently coexists with cancer.^3^, ^4^, ^5^ Cancer survivors face nearly twice the risk of acute coronary syndrome compared with the general population.^5^ A comprehensive understanding of the roles of potential risk factors is crucial for refining cardiovascular risk assessment and management in the rapidly expanding population of cancer survivors.

Importantly, the coexistence of cancer and CHD may partly reflect shared risk factors, including obesity, diabetes, hypertension, hyperlipidemia, tobacco use, diet, alcohol consumption, and physical activity.^6^ Prior evidence from a retrospective study in a Japanese population showed that adherence to Life’s Simple 7, a composite of ideal lifestyle and cardiometabolic factors, was associated with a lower risk of CVD even among cancer survivors.^7^ However, this evidence is limited by its retrospective design and restriction to an Asian population. To date, no study has systematically examined the relationship between the degree of risk factor optimization and CHD risk in White European cancer survivors, nor has its association with residual life expectancy been explored.

Given the clinical relevance of cardiovascular risk management for the growing population of cancer survivors, further investigation is needed to inform evidence-based survivorship care. Therefore, using data from the UK Biobank cohort, we evaluated the associations of joint risk factor control with incident CHD, myocardial infarction (MI), and CVD-related mortality among cancer survivors. To assess cancer-associated excess CHD risk, we compared cancer survivors with matched cancer-free controls. Moreover, we quantified the relative importance of individual risk factors for cardiovascular outcomes and estimated residual life expectancy according to the degree of risk factor control, providing insights for cardiovascular risk stratification and management in cardio-oncology care.

## Methods

### Study design and participants

The UK Biobank is a population-based prospective cohort comprising over 500,000 adults aged 37 to 73 years who were recruited from the United Kingdom between 2006 and 2010. All participants provided written informed consent, and ethical approval was granted by the North West Multi-Centre Research Ethics Committee (reference: 11/NW/0382). The current study utilized data under UK Biobank application ID 91090. At baseline, 23,968 individuals were identified as having a prior diagnosis of cancer, whereas 478,426 participants were classified as cancer-free according to the International Classification of Diseases-10th Revision (ICD-10), codes (C00–C97).^3^ Participants lacking complete risk factor assessments or with pre-existing CHD were excluded. The final analytic sample consisted of 14,557 cancer survivors. To improve comparability between cancer survivors and the reference population while minimizing confounding by major demographic factors, we employed a propensity score–matched design. Cancer survivors were matched to noncancer controls at a 1:4 ratio based on age, sex, and ethnicity. This approach was chosen to balance baseline characteristics between groups and to emulate a pseudo-cohort with comparable distributions of key confounders, thereby improving internal validity.^8^, ^9^, ^10^ This yielded a total study population of 72,785 participants (Supplemental Figure 1).

### Definitions of joint risk factor control

In accordance with European Society of Cardiology guidelines^11^ for CVD prevention and established evidence on CVD risk,^12^, ^13^, ^14^ we assessed 9 modifiable risk factors at baseline (2006-2010). The degree of joint risk factor control was quantified as the total number of risk factors within predefined target ranges. Each risk factor was weighted equally to provide a simple and clinically interpretable measure of overall risk factor control, consistent with prior cardiovascular risk assessment approaches.^7^^,^^15^ Risk factor control was defined as follows: 1) lipid control: low-density lipoprotein cholesterol <2.6 mmol/L (100 mg/dL);^16^ 2) glycemic control: hemoglobin A1c (HbA1c) <48 mmol/mol (6.5%);^17^ 3) blood pressure control: systolic blood pressure <140 mm Hg and diastolic blood pressure <90 mm Hg;^18^ 4) body mass index (BMI) control: 18.5 to 24.9 kg/m^2^ (normal range per WHO criteria); 5) smoking control: current nonsmoking status; 6) diet control: diet quality score ≥7 based on 9 cardiovascular health-promoting dietary components (detailed in Supplemental Table 1);^19^ 7) renal function control: estimated glomerular filtration rate (eGFR) ≥90 mL/min/1.73 m^2^ calculated using the Chronic Kidney Disease-Epidemiology Collaboration equation;^20^ 8) physical activity control: achievement of ≥600 metabolic equivalent of task (MET)-minutes per week;^21^ and 9) sleep health control: sleep health score ≥3 on a 5-point scale, with 1 point assigned for each of the following criteria: early chronotype, 7 to 8 hours of nightly sleep duration, absence of frequent insomnia symptoms, no reported snoring, and absence of excessive daytime sleepiness.^12^ Participants were subsequently categorized into 4 groups according to the degree of risk factor control: ≤3, 4, 5, and ≥6 controlled risk factors.

### Outcome assessment

The primary outcome was incident CHD, and the secondary outcomes included incident MI and CVD-related mortality. Incident CHD and MI were ascertained from hospitalization records using ICD-10 codes, with CHD defined as I21–I25 (which includes MI [I21–I22]).^22^ CVD-related mortality was defined using ICD-10 codes I00–I99 from death registry records, obtained from the National Health Service Information Centre.^13^^,^^22^ Follow-up time was calculated from study enrollment to the occurrence of the specific outcome of interest, all-cause death, study withdrawal, or administrative censoring on January 31, 2022, whichever came first.

### Covariate assessment

Comprehensive baseline data, including demographic characteristics, lifestyle factors, family history, and medication use, were collected using standardized touch-screen questionnaires. Demographic variables including age, sex, ethnicity (categorized as White, South Asian, Black, or other), college education status, Townsend deprivation index (TDI), and annual household income (stratified as <£18,000, £18,000-52,000, or >£52,000) were included as covariates. Lifestyle assessment focused on alcohol consumption patterns, which were evaluated using the question “About how often do you drink alcohol?” with responses categorized into 3 groups: daily consumption, 1 to 4 times weekly, and never/rarely. Family history of cancer and CVD was ascertained through participant self-reports regarding illnesses affecting their father, mother, or siblings. Current use of medications, including lipid-lowering, antihypertensive, and antidiabetic agents, was determined based on self-reported medical conditions at baseline. For participants with a cancer history, specific cancer types and history of chemotherapy/radiotherapy exposure were classified according to ICD-10 diagnostic codes as detailed in Supplemental Table 2.

### Statistical analysis

Baseline characteristics were presented as mean ± SD for continuous variables and as frequencies with percentages for categorical variables, stratified by cardiovascular risk factor control levels (≤3, 4, 5, and ≥6 controlled factors) and matched control status. All covariates were treated as categorical variables, and missing data were addressed using a missing indicator approach. Incidence rates for CHD, MI, and CVD-related mortality were calculated as events per 1,000 person-years.

To estimate the associations between risk factor control and cardiovascular outcomes, we used multivariable Cox proportional hazards models to calculate HRs and 95% CIs for CHD, MI, and CVD-related mortality. The degree of risk factor control was modeled both categorically and continuously. In categorical analyses among cancer survivors, participants with ≤3 controlled risk factors served as the reference group, with adjustment for age, sex, ethnicity, TDI, alcohol consumption, household income, college education, chemotherapy history, radiotherapy history, cancer type, family history of cancer, or CVD. We further assessed cardiovascular risk among cancer survivors relative to matched noncancer controls across different levels of risk factor control, applying the same covariate adjustments except for cancer type and chemotherapy/radiotherapy history. The proportional hazards assumption was verified using Schoenfeld residuals, with no violations detected. Survival curves were constructed using the Kaplan-Meier method, and between-group comparisons were performed using the log-rank test. Restricted cubic spline analyses with 3 knots were used to assess potential dose-response relationships between the number of controlled risk factors and outcomes.

To identify the risk factors most informative for cardiovascular outcomes, we evaluated the relative contribution of 9 modifiable risk factors to CHD, MI, and cardiovascular mortality by computing R^2^ values from Cox regression models.^23^, ^24^, ^25^ We also calculated the explainable log-likelihood attributable to each individual risk factor.^25^ In addition, we examined the association between varying degrees of joint risk factor control and residual life expectancy among cancer survivors and compared these estimates with those of matched noncancer controls. Residual life expectancy was estimated using Royston-Parmar flexible parametric survival models implemented with the *stpm2* command in Stata, with age as the underlying time scale and all-cause mortality as the outcome. The baseline log cumulative hazard was modeled using restricted cubic splines with 3 degrees of freedom. Residual life expectancy was estimated as the restricted mean survival time from ages 50 to 100 years, calculated as the area under the model-predicted survival curve conditional on survival at each annual interval.^26^, ^27^, ^28^ Survival beyond the observed follow-up was projected using the fitted flexible parametric survival model. Model fit was evaluated using Akaike information criterion and Bayesian information criterion (Supplemental Table 3), and the robustness of life expectancy estimates was assessed using alternative spline specifications with 4 and 5 degrees of freedom.

We also conducted comprehensive subgroup analyses stratified by sex, age, ethnicity, education level, TDI, cancer types, and chemotherapy/radiotherapy history. To evaluate the consistency of our findings, we implemented 3 sensitivity analyses: 1) exclusion of outcome events occurring within the first 5 years of follow-up to reduce potential reverse causality; 2) application of the Fine-Gray model to account for competing mortality risk; 3) exclusion of initially cancer-free participants who developed cancer during follow-up from the comparative analysis between cancer survivors and matched controls; and 4) addressing missing covariate data using multiple imputation by chained equations. All analyses were performed using R software (version 4.5.0; R Foundation for Statistical Computing, Vienna, Austria), with statistical significance set at 2-sided *P* < 0.05.

## Results

### Baseline characteristics

This analysis included 14,557 cancer survivors and 58,228 matched noncancer controls. Among cancer survivors, the mean (SD) age was 59.97 (7.02) years and 42.14% were males. Compared with matched noncancer controls, cancer survivors had a lower degree of risk factor control (Supplemental Table 4). Table 1 presents baseline characteristics stratified by the extent of multiple risk factor control among cancer survivors and their corresponding controls. Participants with a higher degree of risk factor control had distinct demographic profiles, including younger age, more females, lower daily alcohol intake, a lower prevalence of current smoking, and higher socioeconomic status, as reflected by higher annual income, greater educational attainment, and lower TDI. They also had a lower prevalence of family histories of cancer and CVD. In addition, cancer survivors with a higher degree of risk factor control reported less use of cardiovascular medications, including lipid-lowering, antihypertensive, and glucose-lowering therapies. Similar baseline patterns associated with higher risk factor control were also observed among matched noncancer participants (Supplemental Table 5).

**Table 1:** Baseline Characteristics of Study Group According to the Degree of Risk Factor Control

	Noncancer (n = 58,228)	Degree of Joint Risk Factor Control			
		≤3 Controlled Risk Factors (n = 2,242)	4 Controlled Risk Factors (n = 3,764)	5 Controlled Risk Factors (n = 4,320)	≥6 Controlled Risk Factors (n = 4,231)
Age, y	59.97 ± 7.02	61.72 ± 6.04	61.23 ± 6.42	60.06 ± 6.85	57.82 ± 7.61
Male, n (%)	24,540 (42.14%)	1,177 (52.50%)	1817 (48.27%)	1809 (41.88%)	1,332 (31.48%)
Ethnicity, n (%)					
White	56,557 (97.13%)	2,170 (96.79%)	3,653 (97.05%)	4,197 (97.15%)	4,119 (97.35%)
Black	488 (0.84%)	32 (1.43%)	36 (0.96%)	32 (0.74%)	22 (0.52%)
South Asian	388 (0.67%)	14 (0.62%)	26 (0.69%)	26 (0.60%)	31 (0.73%)
Other	795 (1.37%)	26 (1.16%)	49 (1.30%)	65 (1.50%)	59 (1.39%)
Cancer type, n (%)					
Breast cancer	-	559 (24.93%)	946 (25.13%)	1,233 (28.54%)	1,354 (32.00%)
Genital urinary cancer	-	655 (29.21%)	949 (25.21%)	951 (22.01%)	745 (17.61%)
Skin cancer	-	475 (21.19%)	944 (25.08%)	1,060 (24.54%)	1,101 (26.02%)
Digestive cancer	-	255 (11.37%)	403 (10.71%)	454 (10.51%)	409 (9.67%)
Hematologic cancer	-	146 (6.51%)	252 (6.70%)	294 (6.81%)	290 (6.85%)
Respiratory cancer	-	47 (2.10%)	69 (1.83%)	67 (1.55%)	57 (1.35%)
Other cancer	-	105 (4.68%)	201 (5.34%)	261 (6.04%)	275 (6.50%)
TDI					
≥ median	27,753 (47.66%)	1,167 (52.05%)	1877 (49.87%)	2,067 (47.85%)	2,016 (47.65%)
< median	30,405 (52.22%)	1,074 (47.90%)	1884 (50.05%)	2,247 (52.01%)	2,209 (52.21%)
Missing	70 (0.12%)	1 (0.04%)	3 (0.08%)	6 (0.14%)	6 (0.14%)
Smoking status, n (%)					
Current	5,117 (8.79%)	459 (20.47%)	366 (9.72%)	282 (6.53%)	120 (2.84%)
Previous	21,949 (37.69%)	873 (38.94%)	1,620 (43.04%)	1,785 (41.32%)	1,570 (37.11%)
Never	31,162 (53.52%)	910 (40.59%)	1778 (47.24%)	2,253 (52.15%)	2,541 (60.06%)
College education, n (%)					
Yes	18,798 (32.28%)	558 (24.89%)	1,044 (27.74%)	1,409 (32.62%)	1,653 (39.07%)
No	39,033 (67.03%)	1,664 (74.22%)	2,689 (71.44%)	2,882 (66.71%)	2,545 (60.15%)
Missing	397 (0.68%)	20 (0.89%)	31 (0.82%)	29 (0.67%)	33 (0.78%)
Drinking status, n (%)					
Daily	13,375 (22.97%)	492 (21.94%)	836 (22.21%)	927 (21.46%)	852 (20.14%)
1-4 times/weekly	28,044 (48.16%)	1,011 (45.09%)	1725 (45.83%)	2,104 (48.70%)	2057 (48.62%)
Never/rarely	16,783 (28.82%)	736 (32.83%)	1,202 (31.93%)	1,287 (29.79%)	1,321 (31.22%)
Missing	26 (0.04%)	3 (0.13%)	1 (0.03%)	2 (0.05%)	1 (0.02%)
Household income, n (%)					
>52,000	11,077 (19.02%)	267 (11.91%)	486 (12.91%)	685 (15.86%)	909 (21.48%)
18,000-52,000	26,801 (46.03%)	947 (42.24%)	1,711 (45.46%)	2,020 (46.76%)	2,008 (47.46%)
<18,000	12,562 (21.57%)	695 (31.00%)	1,038 (27.58%)	1,040 (24.07%)	823 (19.45%)
Missing	7,788 (13.38%)	333 (14.85%)	529 (14.05%)	575 (13.31%)	491 (11.60%)
Sleep quality score	2.85 ± 1.08	1.93 ± 0.91	2.55 ± 1.09	2.94 ± 1.01	3.31 ± 0.88
BMI, kg/m^2^	27.24 ± 4.57	30.12 ± 4.70	28.72 ± 4.33	27.12 ± 4.40	24.51 ± 3.65
Physical activity (MET-minutes/week)	2,695.51 ± 2,678.26	1,585.70 ± 2,204.13	2,526.58 ± 2,655.59	2,705.54 ± 2,567.13	2,976.24 ± 2,574.89
Systolic blood pressure, mm Hg	142.17 ± 20.03	151.95 ± 17.40	147.77 ± 19.04	141.91 ± 19.30	131.35 ± 17.68
Diastolic blood pressure, mm Hg	82.27 ± 10.59	87.03 ± 10.08	85.01 ± 10.37	82.34 ± 9.97	77.31 ± 9.53
LDL-C, mmol/L	3.60 ± 0.87	3.71 ± 0.84	3.68 ± 0.87	3.61 ± 0.89	3.41 ± 0.90
HbA1c, %	36.25 ± 6.20	38.75 ± 8.63	36.90 ± 6.37	36.03 ± 5.38	34.95 ± 4.31
eGFR, mL/min/1.73 m^2^	88.32 ± 12.78	79.79 ± 14.45	83.82 ± 14.59	88.71 ± 13.32	93.77 ± 11.53
Diet score	4.57 ± 1.41	4.09 ± 1.32	4.36 ± 1.30	4.55 ± 1.34	5.00 ± 1.49
Family history of cancer, n (%)	21,584 (37.07%)	967 (43.13%)	1,600 (42.51%)	1793 (41.50%)	1765 (41.72%)
Family history of CVD, n (%)	34,424 (59.12%)	1,355 (60.44%)	2,254 (59.88%)	2,520 (58.33%)	2,372 (56.06%)
Lipid-lowering medication, n (%)	12,328 (21.17%)	637 (28.41%)	893 (23.72%)	852 (19.72%)	638 (15.08%)
Antihypertensive medication, n (%)	14,250 (24.47%)	864 (38.54%)	1,197 (31.80%)	1,098 (25.42%)	659 (15.58%)
Antidiabetes medication, n (%)	2,035 (3.49%)	193 (8.61%)	149 (3.96%)	144 (3.33%)	66 (1.56%)
Chemotherapy, n (%)	-	3,693 (6.34%)	761 (33.94%)	1,153 (30.63%)	1,390 (32.18%)
Radiotherapy, n (%)	-	1,590 (2.73%)	476 (21.23%)	704 (18.70%)	785 (18.17%)

Values are n (%), or mean ± SD.

BMI = body mass index; CVD = cardiovascular diseases; eGFR = estimated glomerular filtration rate; HbA1c = hemoglobin A1c; LDL-C = low-density lipoprotein cholesterol; MET = metabolic equivalent; TDI = Townsend deprivation index.

### Degree of risk factor control and incident CHD, MI, and CVD-related mortality among cancer survivors

Over a median follow-up period of 12.60 years (IQR: 11.78-13.35), we documented 1,298 CHD events, 348 MI events, and 211 CVD-related deaths among cancer survivors, corresponding to incidence rates of 7.93, 2.06, and 1.24 per 1,000 person-years, respectively. Restricted cubic spline analysis demonstrated a significant inverse dose-response relationship between the number of controlled risk factors and the incidence of CHD, MI, and CVD-related mortality (Supplemental Figure 2). After multivariable adjustment, each additional controlled risk factor was associated with a 15% reduction in CHD risk (HR: 0.85, 95% CI: 0.81-0.88), a 19% reduction in MI risk (HR: 0.81, 95% CI: 0.74-0.88), and a 25% reduction in CVD mortality risk (HR: 0.75, 95% CI: 0.67-0.84). Participants with ≥6 controlled risk factors had the lowest risks: 47% lower for CHD (HR: 0.53; 95% CI: 0.44-0.63), 59% lower for MI (HR: 0.41; 95% CI: 0.29-0.58), and 63% lower for CVD-related mortality (HR: 0.37; 95% CI: 0.23-0.58) (Table 2), with a similar pattern observed among noncancer participants (Supplemental Table 6).

**Table 2:** Associations Between the Degree of Joint Risk Factor Control and Risk of Coronary Heart Disease, Myocardial Infarction, and CVD-Related Mortality Among Cancer Survivors

Degree of Joint Risk Factor Control	No. of Cases/Total	IR, per 1,000 Person-Years	Unadjusted Model HR (95% CI), *P* Value	Multivariable Adjusted Model HR (95% CI), *P* Value
CHD				
Overall	1,298/14,557	7.93	–	–
≤3 controlled risk factors	307/2,242	12.93	Reference (1.00)	Reference (1.00)
4 controlled risk factors	407/3,764	9.77	0.75 (0.65-0.87), <0.001	0.82 (0.70-0.95), 0.008
5 controlled risk factors	363/4,320	7.43	0.57 (0.49-0.67), <0.001	0.71 (0.61-0.83), <0.001
≥6 controlled risk factors	221/4,231	4.48	0.35 (0.29-0.41), <0.001	0.53 (0.44-0.63), <0.001
Per 1 additional controlled risk factor	–	–	0.75 (0.72-0.79), <0.001	0.85 (0.81-0.88), <0.001
MI				
Overall	348/14,557	2.06	–	–
≤3 controlled risk factors	91/2,242	3.64	Reference (1.00)	Reference (1.00)
4 controlled risk factors	103/3,764	2.37	0.65 (0.49-0.86), 0.003	0.70 (0.53-0.93), 0.013
5 controlled risk factors	103/4,320	2.05	0.56 (0.42-0.74), <0.001	0.67 (0.51-0.90), 0.007
≥6 controlled risk factors	51/4,231	1.02	0.28 (0.20-0.39), <0.001	0.41 (0.29-0.58), <0.001
Per 1 additional controlled risk factor	–	–	0.73 (0.67-0.79), <0.001	0.81 (0.74-0.88), <0.001
CVD-related mortality				
Overall	211/14,557	1.24	–	–
≤3 controlled risk factors	64/2,242	2.52	Reference (1.00)	Reference (1.00)
4 controlled risk factors	69/3,764	1.57	0.62 (0.44-0.87), 0.005	0.69 (0.49-0.97), 0.034
5 controlled risk factors	50/4,320	0.98	0.39 (0.27-0.56), <0.001	0.51 (0.35-0.74), <0.001
≥6 controlled risk factors	28/4,231	0.55	0.22 (0.14-0.34), <0.001	0.37 (0.23-0.58), <0.001
Per one additional controlled risk factor	–	–	0.65 (0.59-0.73), <0.001	0.75 (0.67-0.84), <0.001

CHD = coronary heart disease; IR = incidence rate; MI = myocardial infarction; other abbreviation as in Table 1.

Multivariable model was adjusted for age, sex, ethnicity (White, South Asian, Black, and other), TDI (≥ median, < median, missing), drinking status (daily,1-4 times/weekly, never/rarely, missing), household income (>£52,000, £18,000-52000, <£18,000, and missing), college education (yes/no, missing), chemotherapy (yes/no), radiotherapy (yes/no), cancer type (hematologic cancer, respiratory cancer, breast cancer, genital urinary cancer, digestive cancer, skin cancer, and other cancer), family history of cancer (yes/no), family history of cardiovascular disease (yes/no).

**Figure 1:**
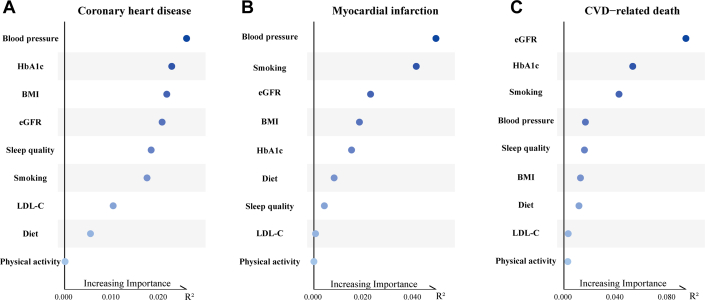
Relative Importance of Risk Factors for Coronary Heart Disease, Myocardial Infarction, and CVD-Related Mortality Among Cancer Survivors BMI = body mass index; CVD = cardiovascular disease; eGFR = estimated glomerular filtration rate; HbA1c = Hemoglobin A1c; LDL-C = low-density lipoprotein cholesterol.

Figure 1 depicts the relative importance of 9 modifiable risk factors for cardiovascular outcomes among cancer survivors, as quantified by explained relative risk (R^2^) models. The hierarchy of risk factor importance varied across outcomes. For CHD, the 5 most influential factors were blood pressure, HbA1c, BMI, eGFR, and sleep quality. For MI, the most informative factors were blood pressure, smoking status, eGFR, BMI, and HbA1c, whereas CVD-related mortality was predominantly influenced by eGFR, HbA1c, smoking status, blood pressure, and sleep quality. These findings were supported by explained log-likelihood analysis (Supplemental Figure 3).

Cancer type-specific analyses revealed that hematologic and respiratory malignancies were associated with the highest risk for adverse cardiovascular outcomes compared with noncancer controls (Supplemental Table 7). Subgroup analyses suggested that the association between joint risk factor control and lower CHD risk was more pronounced among cancer survivors younger than 65 years than among older survivors (*P* for interaction = 0.020) (Supplemental Table 8). In addition, the association between joint risk factor control and lower CVD-related mortality was more evident among cancer survivors who had not received chemoradiotherapy (*P* for interaction = 0.010) (Supplemental Table 10). No significant interactions were observed for sex, ethnicity, TDI, or cancer type in the associations between joint risk factor control and cardiovascular outcomes (Supplemental Tables 8 to 10). Sensitivity analyses yielded results consistent with the primary findings (Supplemental Tables 11 to 13).

### Degree of risk factor control and incident CHD, MI, and CVD-related mortality among cancer survivors compared with noncancer controls

**Figure 2:**
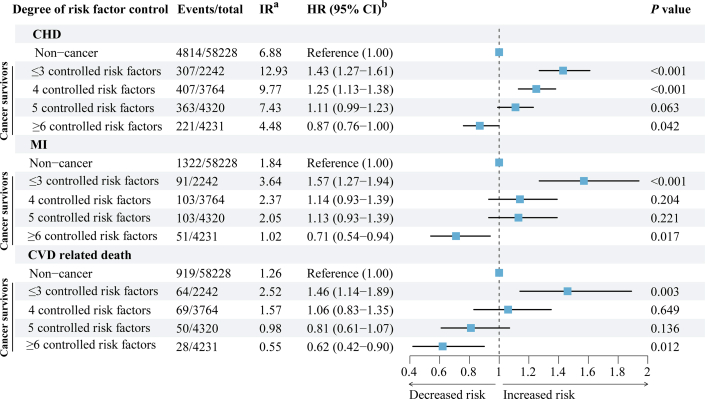
Degree of Risk Factor Control and Risks of CHD, MI, and CVD-Related Mortality Among Cancer Survivors Compared With Matched Noncancer Controls ^a^per 1,000 person-years; ^b^Models were adjusted for age, sex, ethnicity (White, South Asian, Black, and other), TDI (≥ median, < median, missing), drinking status (daily, 1-4 times/weekly, never/rarely, missing), household income (>£52,000, £18,000-52000, <£18,000, and missing), college education (yes/no, missing), family history of cancer (yes/no), family history of cardiovascular disease (yes/no). CHD = coronary heart disease; MI = myocardial infarction; IR = incidence rate; other abbreviation as in Figure 1.

To examine whether cancer-related excess risk of CHD, MI, and CVD-related mortality varied according to the degree of joint risk factor control, we evaluated cardiovascular event incidence across categories of controlled risk factors among cancer survivors and their matched noncancer controls (Figure 2, Central Illustration). In multivariable-adjusted analyses, each additional risk factor controlled within target ranges was associated with progressively lower risks of CHD, MI, and CVD-related mortality among cancer survivors relative to noncancer controls (all *P* for trend <0.001). Cancer survivors with ≤3 controlled risk factors had the highest cardiovascular risks, with HRs of 1.43 (95% CI: 1.27-1.61) for CHD, 1.57 (95% CI: 1.27-1.94) for MI, and 1.46 (95% CI: 1.14-1.89) for CVD-related mortality. Importantly, when 5 or more risk factors were adequately controlled, the excess CHD risk associated with cancer history was no longer statistically significant compared with matched controls (HR: 1.11, 95% CI: 0.99-1.23). For MI and CVD-related mortality, the excess risks were no longer statistically significant among cancer survivors with ≥4 controlled risk factors (HR: 1.14; 95% CI: 0.93-1.39 and HR: 1.06; 95% CI: 0.83-1.35, respectively). Kaplan-Meier survival analyses showed consistent patterns (Supplemental Figure 4).

**Figure 3:**
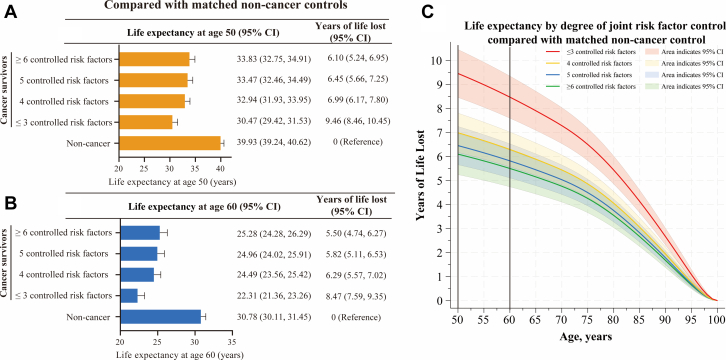
Residual Life Expectancy by Degree of Risk Factor Control Among Cancer Survivors Compared With Matched Noncancer Controls

**Central Illustration:**
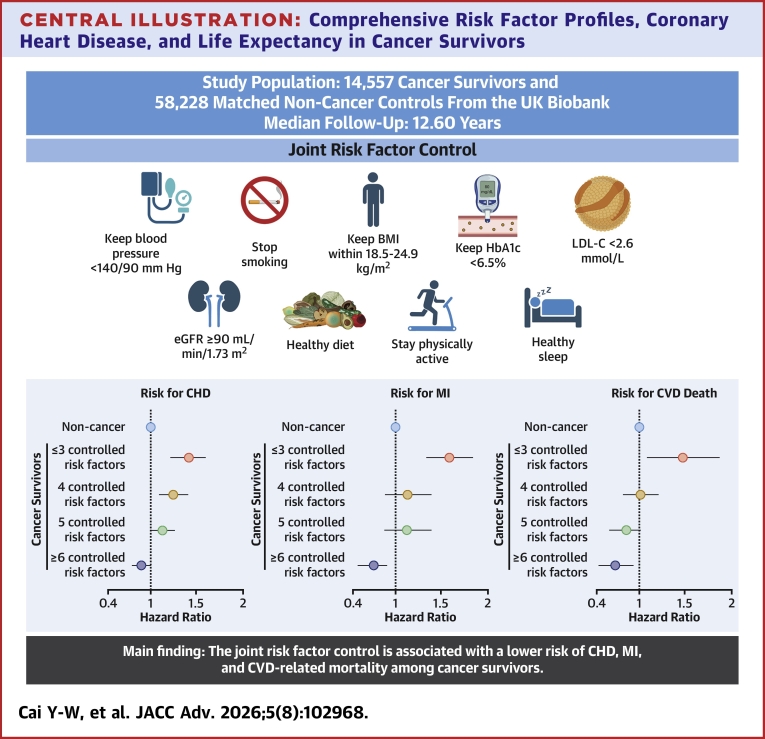
Comprehensive Risk Factor Profiles, Coronary Heart Disease, and Life Expectancy in Cancer Survivors BMI = body mass index; CHD = coronary heart disease; CVD = cardiovascular disease; eGFR = estimated glomerular filtration rate; HbA1c = Hemoglobin A1c; LDL-C = low-density lipoprotein cholesterol; MI = myocardial infarction.

When evaluating the joint association of cancer status and degree of risk factor control with incident CHD, MI, and CVD-related mortality, we used noncancer participants with control of ≥6 risk factors as the reference group. Within this framework, cancer survivors with the lowest degree of risk factor control had the greatest risks of CHD, MI, and CVD-related mortality, with HRs of 2.11 (95% CI: 1.85-2.40), 2.75 (95% CI: 2.14-3.53), and 2.22 (95% CI: 1.65-2.98), respectively (Supplemental Figure 5).

The robustness of these results was validated through a series of sensitivity analyses, including exclusion of events occurring within the first 5 years of follow-up, competing risk analysis accounting for noncardiovascular death, multiple imputation for missing covariates, and exclusion of participants who developed cancer during the follow-up period (Supplemental Tables 14-17).

### Degree of risk factor control and residual life expectancy

We further examined the association between the degree of joint risk factor control and differences in residual life expectancy among cancer survivors compared with matched noncancer controls (Figure 3). Among cancer survivors, those with ≤3 controlled risk factors had the shortest residual life expectancy, with 30.47 years (95% CI: 29.42-31.53) at age 50 and 22.31 years (95% CI: 21.36-23.26) at age 60. Residual life expectancy was progressively longer with a greater number of controlled risk factors. Compared with matched noncancer controls, cancer survivors had significantly shorter life expectancy across all risk factor control categories, with the largest deficit observed among those with ≤3 controlled risk factors. At age 50, this group experienced 9.46 years of life lost (95% CI: 8.46-10.45 years). Notably, a higher degree of joint risk factor control was associated with a smaller cancer-related life expectancy deficit. Cancer survivors with ≥6 controlled risk factors had an attenuated life expectancy deficit of 6.10 years (95% CI: 5.24-6.95 years) at age 50 compared with matched noncancer controls. Similar life expectancy estimates were observed across alternative spline specifications with 4 and 5 degrees of freedom, indicating robust findings (Supplemental Table 18).

## Discussion

In this large prospective cohort study, a higher degree of joint risk factor control was significantly associated with lower risks of CHD, MI, CVD-related mortality, as well as longer residual life expectancy among cancer survivors. Each additional controlled risk factor was associated with a 15% to 25% lower risk of these cardiovascular outcomes. These findings highlight the clinical relevance of comprehensive assessment and management of cardiovascular risk factor in this vulnerable population.

Cancer survivors have been consistently reported to experience higher risks of CHD and MI than cancer-free individuals, likely reflecting shared risk factors, cancer-related biological processes, and treatment-associated cardiovascular effects.^3^, ^4^, ^5^ Current cardiovascular health frameworks, including Life’s Simple 7^7^ and the more recent Life’s Essential 8^29^, have established the importance of integrated risk factor management in reducing CVD risk. The multiple risk factor control strategy adopted in our study is conceptually aligned with these frameworks, as all highlight the cumulative relevance of modifiable lifestyle and metabolic factors. However, rather than proposing a novel scoring construct, our study addresses a distinct and clinically relevant question that has not been adequately explored in prior literature:^7^^,^^29^ among cancer survivors with optimal control of multiple cardiovascular risk factors, to what extent do excess CHD risk and remaining life expectancy approach the levels observed in cancer-free individuals? By incorporating a matched noncancer reference population, our analysis extends beyond within-cohort comparisons and evaluates how differences in the risks of CHD, MI, and CVD-related mortality between cancer survivors and noncancer controls vary according to the degree of risk factor control. This comparative framework enables a more clinically meaningful assessment that cannot be inferred from studies restricted to cancer survivors alone. Specifically, our findings showed that excess CHD risk was no longer evident among cancer survivors with ≥5 risk factors controlled within target ranges. In addition, differences in MI and CVD-related mortality between cancer survivors and matched cancer-free controls were smaller among survivors with at least 4 controlled risk factors.

Among the individual components, blood pressure, HbA1c, BMI, eGFR, sleep quality, and smoking were the most informative factors for predicting CHD, MI, and CVD-related mortality among cancer survivors. Blood pressure showed the strongest contribution to CHD and MI risk prediction, whereas eGFR was most strongly associated with CVD-related mortality. These findings are consistent with prior studies in the general population highlighting the central role of blood pressure in CHD risk^30^ and the prognostic value of eGFR for cardiovascular mortality.^31^ These results suggest that attention to these specific factors may be particularly relevant when assessing cardiovascular risk among cancer survivors.

The association between joint risk factor control and lower CHD risk appeared more pronounced among cancer survivors younger than 65 years. As aging is a shared risk determinant for both malignancy and CHD, older cancer patients inherently face elevated cardiovascular vulnerability,^32^ which could partly explain the weaker association observed in this subgroup. For CVD-related mortality, the association with joint risk factor control appeared more evident among cancer survivors who had not received chemoradiotherapy. This difference may be related to treatment-associated cardiotoxicity and heart failure, which may contribute to higher CVD-related mortality and reduce the relative benefits of risk factor modification.^33^

To our knowledge, this study is among the first to examine the association between comprehensive risk factor control and residual life expectancy among cancer survivors. Consistent with previous research demonstrating increased mortality among cancer patients,^1^ we observed 6.10 to 9.46 fewer life-years at age 50 among cancer survivors compared with matched noncancer controls. Within this context, a higher degree of controlled risk factors was associated with longer residual life expectancy among cancer survivors. These findings suggest that comprehensive assessment and optimization of multiple modifiable risk factors may be clinically relevant for long-term health management in cancer survivorship care.

The mechanisms underlying the cumulative association between joint risk factor control and lower CHD risk among cancer survivors remain to be clarified. Cancer and CHD share a broad range of traditional risk factors, and cancer-related processes or treatments may further amplify exposure to cardiometabolic hazards. For example, vascular endothelial growth factor inhibitors and tyrosine kinase inhibitors are associated with an increased risk of hypertension among treated patients.^34^ Corticosteroids and immune checkpoint inhibitors also disrupt glucose homeostasis and foster insulin resistance, adding an additional metabolic burden.^35^^,^^36^ These cancer-specific perturbations, superimposed on conventional cardiometabolic risk factors, provide a plausible biological explanation for why joint control of multiple modifiable risks may be associated with lower cardiovascular risk among cancer survivors. Moreover, multiple risk factor control may synergistically alleviate metabolic derangements, oxidative stress, neurohormonal dysregulation, and inflammation, all of which have been implicated in cancer-related CHD.^6^

### Study limitations

Although our study benefits from a large sample of cancer survivors from the UK Biobank and extensive covariate information, several limitations should be acknowledged. First, because of the observational design, risk factors were assessed only at baseline, and longitudinal changes or interventions during follow-up could not be evaluated. This limits characterization of dynamic risk factor trajectories and precludes causal inference. Second, we were unable to ascertain the timing of cancer diagnosis or treatment relative to study enrollment; therefore, heterogeneity in survivorship duration could not be fully addressed and may have introduced residual bias. Third, detailed treatment information, including treatment type, cumulative exposure, and timing was unavailable, limiting evaluation of treatment-specific cardiotoxicity and potential interactions with risk factor control. Fourth, several variables used for risk factor assessment, including smoking, diet, physical activity, and sleep characteristics, were self-reported and may be subject to recall bias and misclassification. Fifth, uniform thresholds for systolic blood pressure and low-density lipoprotein cholesterol may not fully reflect individualized guideline-recommended targets across different risk profiles, potentially resulting in misclassification of risk factor control. Finally, because most participants were White individuals from the United Kingdom, the generalizability of these findings to more diverse racial, ethnic, and geographic populations requires further study.

## Conclusions

This prospective study demonstrates an inverse relationship between the degree of joint risk factor control and incident CHD, MI, and CVD-related mortality among cancer survivors. These findings support the clinical relevance of comprehensive risk factor assessment and optimization in cancer survivorship programs for cardiovascular risk management in this vulnerable population.

Perspectives **COMPETENCY IN MEDICAL KNOWLEDGE:** Among cancer survivors, a higher degree of joint control of modifiable cardiovascular risk factors was associated with lower risks of CHD, MI, and CVD-related mortality, as well as longer residual life expectancy. Excess CHD risk was not evident among survivors with ≥5 controlled risk factors compared with matched cancer-free controls, whereas differences in MI and CVD-related mortality were smaller among those with ≥4 controlled risk factors.

**TRANSLATIONAL OUTLOOK:** Comprehensive assessment of modifiable cardiovascular risk factors may help refine cardio-oncology risk stratification and survivorship care. Future studies are needed to determine whether sustained risk factor optimization is associated with improved long-term cardiovascular outcomes among cancer survivors.

## Funding support and author disclosures

This work was supported by the 10.13039/100014717National Natural Science Foundation of China (grant number: 82070237 and 82170457). The funders had no role in the study design, data collection and analysis, publication decision, or manuscript preparation. The authors have reported that they have no relationships relevant to the contents of this paper to disclose.
