# Supplementary material for: Comprehensive Risk Factor Profiles, Coronary Heart Disease, and Life Expectancy in Cancer Survivors
Source: JACC Adv. 2026 Jul 17;5(8):102968. doi: 10.1016/j.jacadv.2026.102968 (PMC13400872; doi:10.1016/j.jacadv.2026.102968)
Supplement: Supplementary Materials [file mmc1.docx]

**Comprehensive Risk Factor Profiles, Coronary Heart Disease, and Life Expectancy in Cancer Survivors**

Author list: Yang-Wei Cai, Chuan-Rui Zeng, Mao-Xiong Wu, Yi Zhang, Pin-Ming Liu, Jing-Feng Wang, Jing-Wei Gao, Hai-Feng Zhang, Yang-Xin Chen

Categories

[Supplemental Figure 1. Study flowchart. 3](#_Toc231245314)

[Supplemental Figure 2. Restricted cubic spline curves showed the association between number of controlled risk factors and CHD, MI, and CVD–related death 4](#_Toc231245315)

[Supplemental Figure 3. Relative importance of risk factors for preventing CHD, MI, and CVD–related mortality among cancer survivors using explained log–likelihood analysis 5](#_Toc231245316)

[Supplemental Figure 4. Kaplan–Meier estimates of CHD, MI, and CVD-related mortality according to the number of controlled risk factors among cancer survivors and matched non-cancer controls. 6](#_Toc231245317)

[Supplemental Figure 5. Joint association between cancer and degree of joint risk factor control and risk of incident CHD, MI, and CVD–related mortality. 7](#_Toc231245318)

[Supplemental Table 1. Individual food items and their categorization. 8](#_Toc231245319)

[Supplemental Table 2. ICD–10 diagnostic codes for specific cancer types. 9](#_Toc231245320)

[Supplemental Table 3. Model fit statistics for Royston–Parmar flexible parametric survival models with alternative spline specifications 10](#_Toc231245321)

[Supplemental Table 4. Degree of Joint Risk Factor Control Among Cancer Survivors and Matched Controls. 11](#_Toc231245322)

[Supplemental Table 5. Baseline characteristics of study group according to the degree of risk factor control in non-cancer participants. 12](#_Toc231245323)

[Supplemental Table 6. Associations between the degree of joint risk factor control and risk of CHD, MI, and CVD-related mortality among non–cancer participants. 13](#_Toc231245324)

[Supplemental Table 7. Association between cancer types and risk of incident CHD, MI, and CVD–related mortality. 14](#_Toc231245325)

[Supplemental Table 8. Subgroup analyses: associations between degree of joint risk factor control and risk of incident CHD among cancer survivors. 15](#_Toc231245326)

[Supplemental Table 9. Subgroup analyses: associations between degree of joint risk factor control and risk of incident myocardial infarction among cancer survivors. 16](#_Toc231245327)

[Supplemental Table 10. Subgroup analyses: associations between degree of joint risk factor control and risk of incident CVD-related mortality among cancer survivors. 17](#_Toc231245328)

[Supplemental Table 11. Sensitivity analyses: associations between degree of joint risk factor control and risk of CHD, MI, and CVD-related mortality among cancer survivors after excluding events occurring within the first 5 years of follow–up (n=14,026). 18](#_Toc231245329)

[Supplemental Table 12. Sensitivity analyses: associations between degree of joint risk factor control and risk of CHD, MI, and CVD-related mortality among cancer survivors using Fine & Gray Models for competing risk. 19](#_Toc231245330)

[Supplemental Table 13. Multiple-imputation sensitivity analyses of joint risk factor control and risks of CHD, MI, and CVD-related mortality among cancer survivors. 20](#_Toc231245331)

[Supplemental Table 14. Sensitivity analyses: associations between degree of joint risk factor control and risk of CHD, MI, and CVD-related mortality compared to matched control group after excluding events occurring within the first 5 years of follow–up (n=70,348). 21](#_Toc231245332)

[Supplemental Table 15. Sensitivity analyses: associations between degree of joint risk factor control and risk of coronary heart disease compared to matched control group using Fine & Gray Models for competing risk. 22](#_Toc231245333)

[Supplemental Table 16. Multiple-imputation sensitivity analyses of the associations between joint risk factor control and risks of CHD, MI, and CVD-related mortality among cancer survivors compared with matched non-cancer controls. 23](#_Toc231245334)

[Supplemental Table 17. Sensitivity analyses: associations between degree of joint risk factor control and risk of coronary heart disease following exclusion of initially cancer–free participants who developed cancer during follow–up (n=62,541) 24](#_Toc231245335)

[Supplemental Table 18. Sensitivity analysis of residual life expectancy estimates using alternative spline degrees of freedom in Royston–Parmar flexible parametric survival models 25](#_Toc231245336)

**
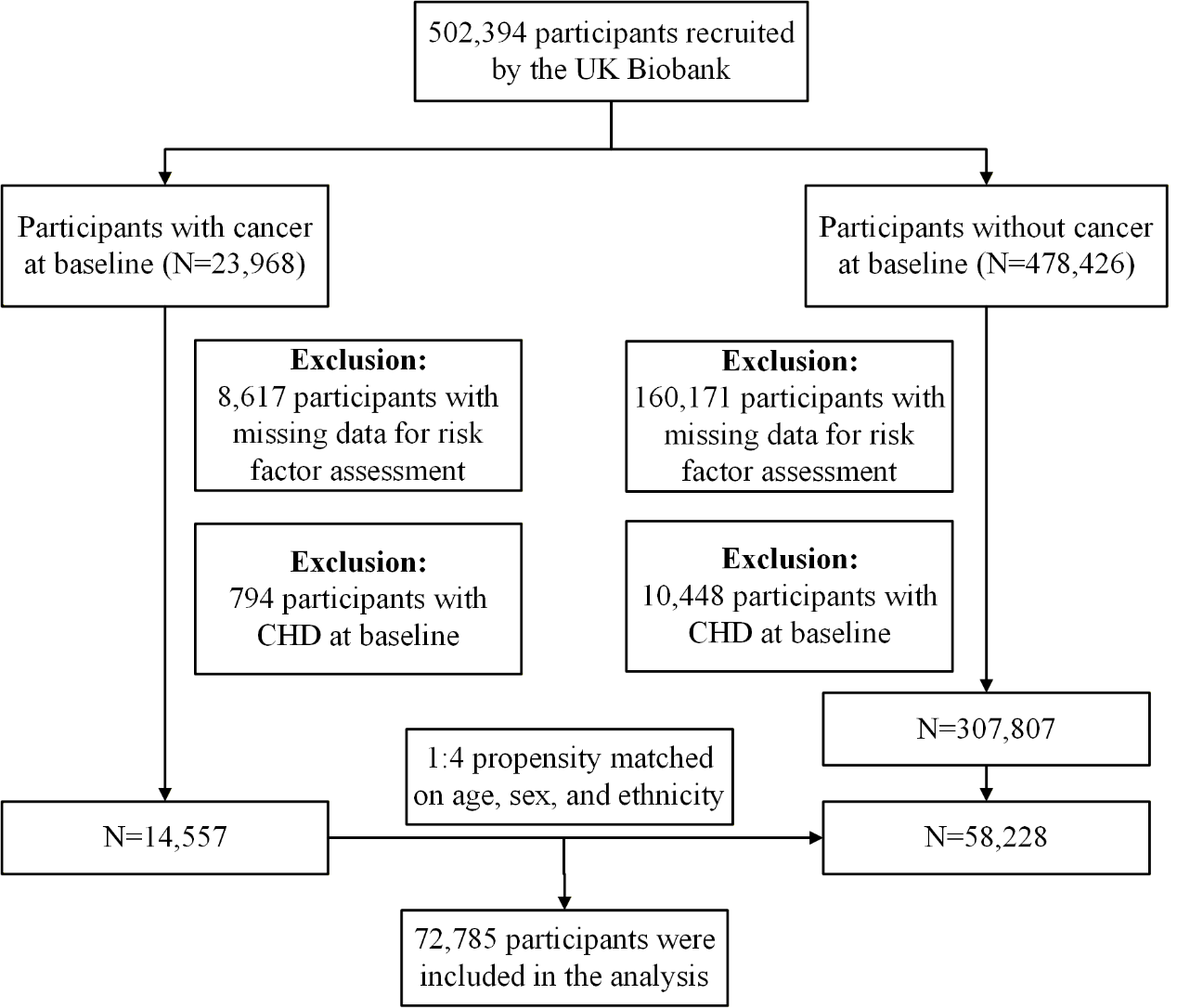
**

# Supplemental Figure 1. Study flowchart.

Abbreviations: CHD, coronary heart disease.


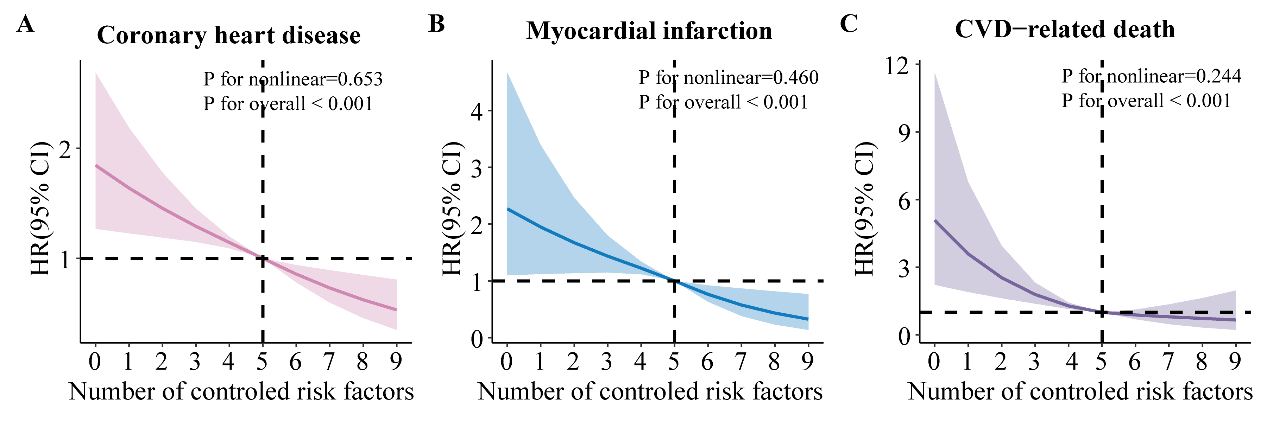
Supplemental Figure 2. Restricted cubic spline curves showed the association between number of controlled risk factors and CHD, MI, and CVD–related death**.**


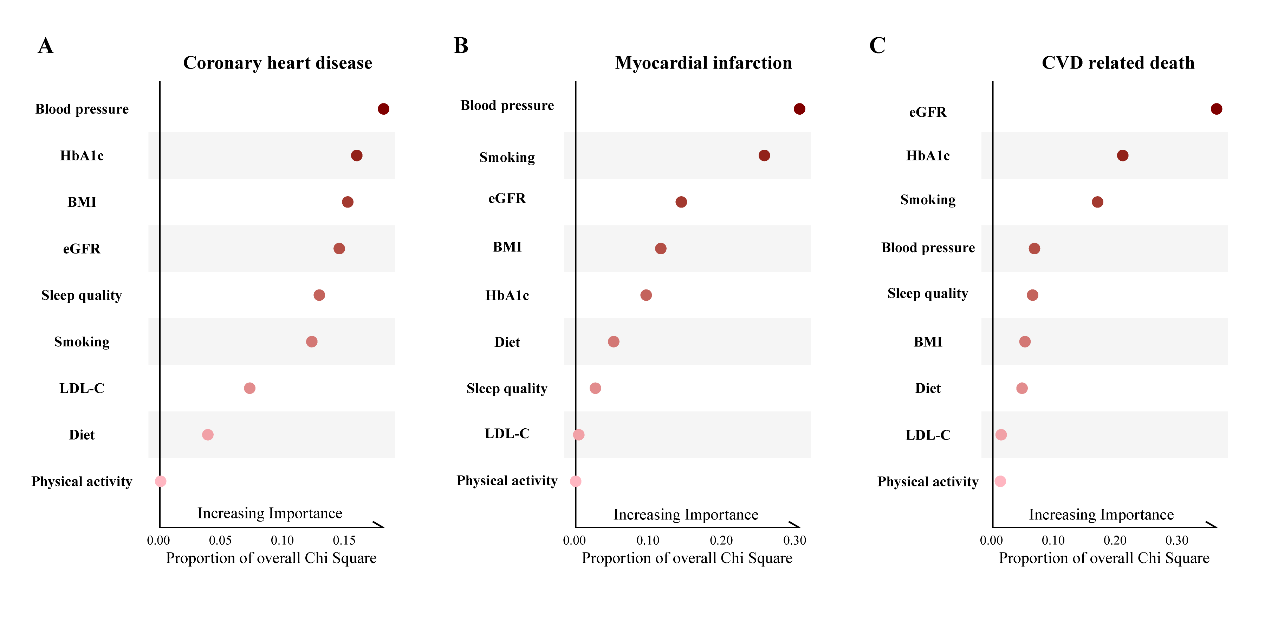


Supplemental Figure 3. Relative importance of risk factors for preventing CHD, MI, and CVD–related mortality among cancer survivors using explained log–likelihood analysis**.** Abbreviations: BMI, body mass index; HbA1c, Hemoglobin A1c; LDL–C, low–density lipoprotein cholesterol; eGFR, estimated glomerular filtration rate.

**
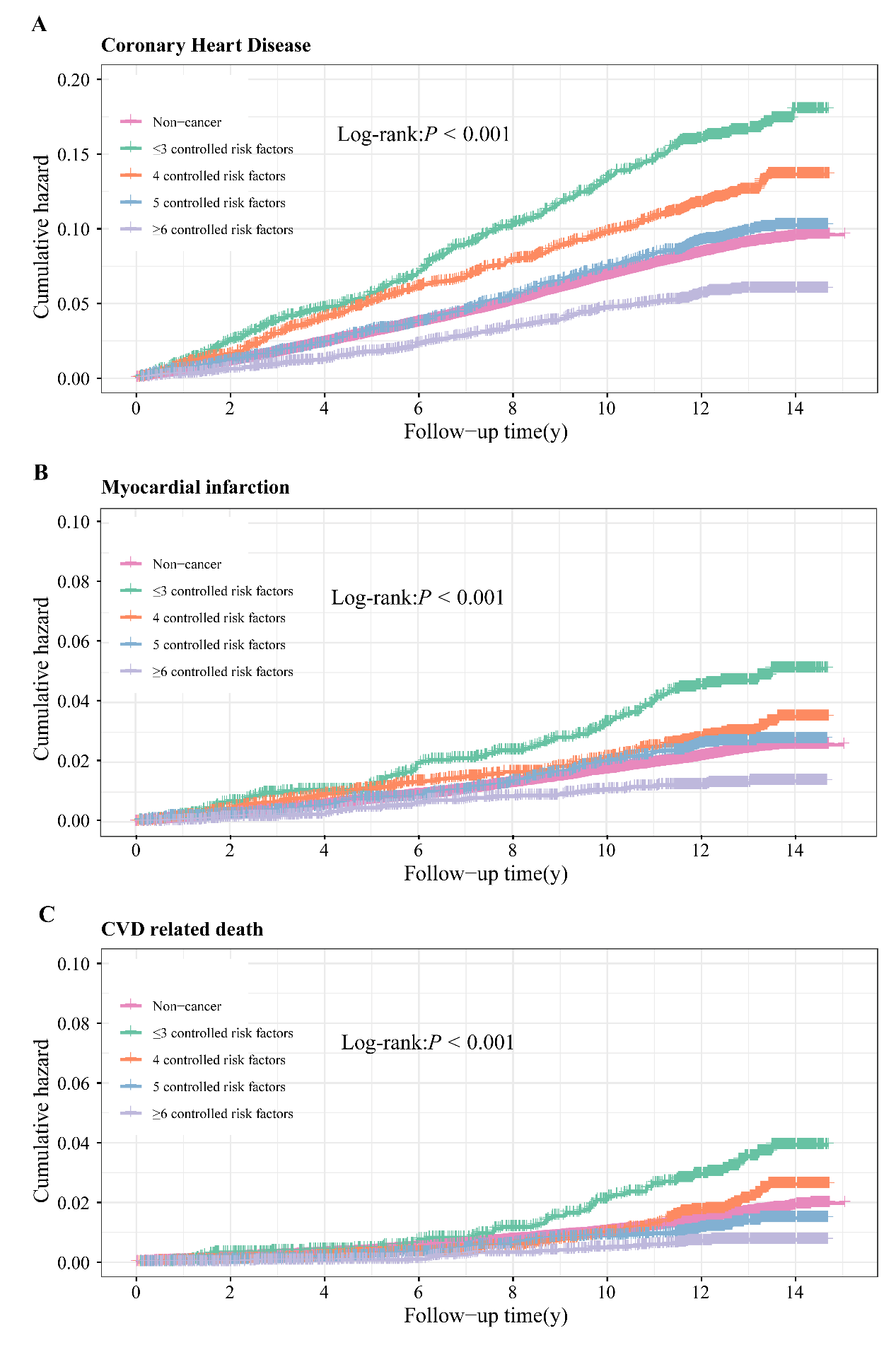
**

# Supplemental Figure **4**. Kaplan–Meier estimates of CHD, MI, and CVD-related mortality according to the number of controlled risk factors among cancer survivors and matched non-cancer controls.

**
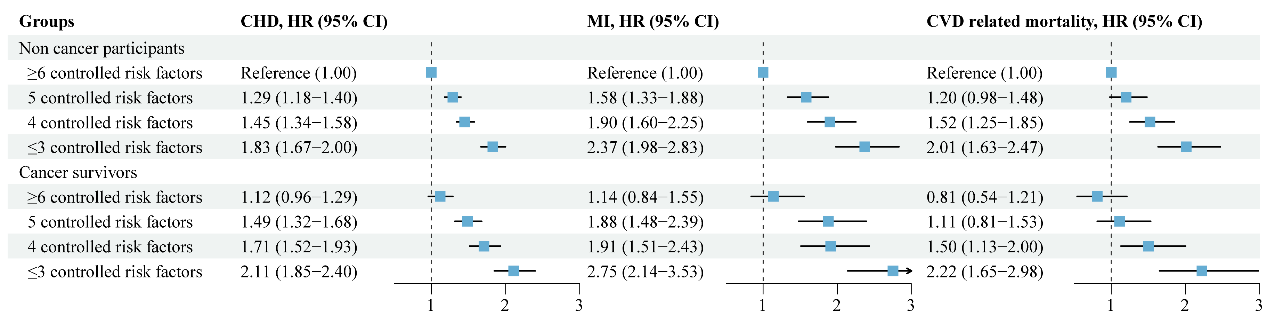
**

Supplemental Figure 5. Joint association between cancer and degree of joint risk factor control and risk of incident CHD, MI, and CVD–related mortality. Adjusted for age, sex, ethnicity (White, South Asian, Black, and other), TDI (≥ median, < median, missing), drinking status (daily,1–4 times/weekly, never/rarely, missing), household income (>£52,000, £18,000–52000, <£18,000, and missing), college education (yes/no, missing), family history of cancer (yes/no), family history of cardiovascular disease (yes/no).

# Supplemental Table 1. Individual food items and their categorization.

| **Variable** | **Categories reported from the touch–screen questionnaire** | **Binary Variables** |
| --- | --- | --- |
| Fruit & vegetables (regrouped from fruit, dried fruit & Vegetable) | Serving/day | ≥5 serving/day (Ref.) <5 serving/day |
| Total fish intake (regrouped from both total non–oily fish and oily fish) | Never less than once a week; Once a week 2–4 times a week; 5–6 times a week; Once or more daily | ≥2 times a week (at least once a week of each category) (Ref.) <once a week of each one |
| Processed meat intake | Never; Less than once a week; Once a week 2–4 times a week; 5–6 times a week; Once or more daily | ≤Once a week (Ref.) >Once a week |
| Red meat (regrouped from beef, pork and lamb) | Never; Less than once a week; Once a week 2–4 times a week; 5–6 times a week; Once or more daily | ≤Once a week (Ref.) >Once a week |
| Milk type used | Full cream; Semi–skimmed; Skimmed; Soya; another type of milk; Never rarely have milk | Semi–skimmed/skimmed (Ref.) Full cream/ another type of milk/ never rarely have milk |
| Spread type | Never/rarely; Butter; Other type/margarine; Flora pro–active/benecol | Never/rarely (Ref.) Another selection |
| Cereal intake | Bowls/week | >5 bowls (Ref) ≤5 bowls |
| Salt added to food | Never/rarely; Sometimes; Usually; Always | Never/rarely (Ref.) Another selection |
| Water intake | Glasses/day | ≥6 glasses (Ref.) <6 glasses |

We assigned 1–point to participants for each healthy category met, defined as more than 5 serving per day of fruits and vegetables; total fish intake more than twice per week; processed meat less than once per week; red meat less than once per week; consumption of semi–skimmed/skimmed milk; never or rarely intake spread; more than 5 bowls per week of cereal; never or rarely add salt to food; more than 6 glasses per day of water; and. Participants’ points were summed to create an unweighted score, with a maximum score of 9 representing the “healthiest” score, and a minimum score of 0 representing the “least healthy” score.

# Supplemental Table 2. ICD–10 diagnostic codes for specific cancer types.

| **Cancer diagnosis** | **ICD–10 codes** |
| --- | --- |
| Breast cancer | C50 |
| Digestive cancer | C15–C26 |
| Respiratory cancer | C30–C39 |
| Skin cancer | C43, C44 |
| Genital and urinary tract cancer | C51–C68 |
| Hematologic/lymphatic cancer | C81–C88, C90–C96 |
| Other cancer type | C0, C10–C14, C40–C41, C45–C49, C69–C80, C97 |
| Chemotherapy | T451, Z082, Z087, Z511, Z512, Z542, Z926 |
| Radiotherapy | T66, Y842, Z081, Z087, Z510, Z541, Z923 |

# Supplemental Table 3. Model fit statistics for Royston–Parmar flexible parametric survival models with alternative spline specifications

| **Degree of freedom** | **Log likelihood** | **AIC** | **BIC** |
| --- | --- | --- | --- |
| 3 | -9038.25 | 18094.51 | 18177.24 |
| 4 | -9041.35 | 18098.70 | 18172.24 |
| 5 | -9037.16 | 18094.33 | 18186.25 |

# Supplemental Table 4. Degree of joint risk factor control among cancer survivors and matched non-cancer controls.

| **Cancer diagnosis** | **Matched non-cancer controls (N=58,228)** | **Cancer survivors (N=14,557)** | ***P* value** |
| --- | --- | --- | --- |
| Degree of joint risk factor control |  |  | 0.010 |
| ≥6 controlled risk factors | 17585 (30.20%) | 4231 (29.07%) |  |
| 5 controlled risk factors | 14901 (25.59%) | 3764 (25.86%) |  |
| 4 controlled risk factors | 17284 (29.68%) | 4320 (29.68%) |  |
| ≤3 controlled risk factors | 8458 (14.53%) | 2242 (15.40%) |  |
| Number of controlled risk factors | 4.85±1.29 | 4.81±1.30 | 0.003 |

Categorical variables are presented as n (%), and continuous variables are presented as mean±SD.

**Supplemental Table 5.** **Baseline characteristics of study group according to the degree of risk factor control in non-cancer participants.**

|  | | Degree of joint risk factor control | | | | | | |
| --- | --- | --- | --- | --- | --- | --- | --- | --- |
|  |  | ≤3 controlled risk factors (n=8,458) | | 4 controlled risk factors (n=14,901) | | 5 controlled risk factors (n=17,284) | | ≥ 6 controlled risk factors (n=17,585) |
| Age, years | | 61.86 ±6.04 | | 61.26 ±6.32 | | 60.02 ±6.87 | | 57.92 ±7.62 |
| Male, n (%) | | 4489 (53.07%) | | 7318 (49.11%) | | 7326 (42.39%) | | 5407 (30.75%) |
| Ethnicity, n (%) | |  | |  | |  | |  |
| White | | 8147 (96.32%) | | 14492 (97.26%) | | 16799 (97.19%) | | 17119 (97.35%) |
| Black | | 135 (1.60%) | | 136 (0.91%) | | 125 (0.72%) | | 92 (0.52%) |
| South Asian | | 56 (0.66%) | | 78 (0.52%) | | 126 (0.73%) | | 128 (0.73%) |
| Other | | 120 (1.42%) | | 195 (1.31%) | | 234 (1.35%) | | 246 (1.40%) |
| TDI | |  | |  | |  | |  |
| ≥ median | | 4249 (50.24%) | | 7284 (48.88%) | | 8100 (46.86%) | | 8120 (46.18%) |
| < median | | 4197 (49.62%) | | 7602 (51.02%) | | 9159 (52.99%) | | 9447 (53.72%) |
| Missing | | 12 (0.14%) | | 15 (0.10%) | | 25 (0.14%) | | 18 (0.10%) |
| Smoking status, n (%) | |  | |  | |  | |  |
| Current | | 1850 (21.87%) | | 1552 (10.42%) | | 1170 (6.77%) | | 545 (3.10%) |
| Previous | | 3124 (36.94%) | | 5909 (39.66%) | | 6649 (38.47%) | | 6267 (35.64%) |
| Never | | 3484 (41.19%) | | 7440 (49.93%) | | 9465 (54.76%) | | 10773 (61.26%) |
| College education, n (%) |  | |  | |  | |  |  |
| Yes | | 2153 (25.46%) | | 4204 (28.21%) | | 5506 (31.86%) | | 6935 (39.44%) |
| No | | 6232 (73.68%) | | 10577 (70.98%) | | 11675 (67.55%) | | 10549 (59.99%) |
| Missing | | 73 (0.86%) | | 120 (0.81%) | | 103 (0.60%) | | 101 (0.57%) |
| Drinking status, n (%) | |  | |  | |  | |  |
| Daily | | 1935 (22.88%) | | 3570 (23.96%) | | 4013 (23.22%) | | 3857 (21.93%) |
| 1–4 times/weekly | | 3824 (45.21%) | | 7098 (47.63%) | | 8407 (48.64%) | | 8715 (49.56%) |
| Never/rarely | | 2698 (31.90%) | | 4225 (28.35%) | | 4853 (28.08%) | | 5007 (28.47%) |
| Missing | | 1 (0.01%) | | 8 (0.05%) | | 11 (0.06%) | | 6 (0.03%) |
| Household income, n (%) |  | |  | |  | |  |  |
| >52,000 | | 1171 (13.84%) | | 2402 (16.12%) | | 3319 (19.20%) | | 4185 (23.80%) |
| 18,000–52,000 | | 3678 (43.49%) | | 6910 (46.37%) | | 8075 (46.72%) | | 8138 (46.28%) |
| <18,000 | | 2376 (28.09%) | | 3544 (23.78%) | | 3599 (20.82%) | | 3043 (17.30%) |
| Missing | | 1233 (14.58%) | | 2045 (13.72%) | | 2291 (13.26%) | | 2219 (12.62%) |
| Sleep quality score | | 1.99 ±0.94 | | 2.61 ±1.08 | | 2.98 ±1.02 | | 3.34 ±0.87 |
| BMI, kg/m^2^ | | 30.10 ±4.61 | | 28.82 ±4.30 | | 27.20 ±4.21 | | 24.56 ±3.58 |
| Physical activity (MET–minutes/week) | | 1750.09 ±2416.44 | | 2577.97 ±2685.06 | | 2850.47 ±2658.21 | | 3097.53 ±2695.04 |
| Systolic blood pressure, mmHg | | 151.91 ±17.29 | | 148.53 ±19.02 | | 142.33 ±19.65 | | 131.92 ±17.70 |
| Diastolic blood pressure, mmHg | | 87.06 ±10.13 | | 85.25 ±10.33 | | 82.32 ±10.18 | | 77.39 ±9.33 |
| LDL–C, mmol/L | | 3.71 ±0.82 | | 3.71 ±0.85 | | 3.64 ±0.86 | | 3.43 ±0.88 |
| HbA1c, % | | 38.77 ±9.07 | | 36.84 ±6.42 | | 35.94 ±5.38 | | 34.85 ±4.33 |
| eGFR, mL/min/1.73m^2^ | | 81.31 ±12.79 | | 84.68 ±12.77 | | 88.97 ±12.29 | | 94.13 ±10.33 |
| Diet score | | 4.11 ±1.32 | | 4.34 ±1.31 | | 4.55 ±1.35 | | 5.02 ±1.47 |
| Family history of cancer, n (%) | | 3230 (38.19%) | | 5663 (38.00%) | | 6425 (37.17%) | | 6266 (35.63%) |
| Family history of CVD, n (%) | | 5106 (60.37%) | | 8952 (60.08%) | | 10328 (59.75%) | | 10038 (57.08%) |
| Lipid–lowering medication, n (%) | | 2436 (28.80%) | | 3512 (23.57%) | | 3549 (20.53%) | | 2831 (16.10%) |
| Anti–hypertensive medication, n (%) | | 3020 (35.71%) | | 4421 (29.67%) | | 4124 (23.86%) | | 2685 (15.27%) |
| Anti–diabetes medication, n (%) | | 651 (7.70%) | | 572 (3.84%) | | 480 (2.78%) | | 332 (1.89%) |

Categorical variables are presented as n (%), and continuous variables are presented as mean±SD. Abbreviations: CVD, cardiovascular diseases; eGFR, estimated glomerular filtration rate; LDL–C, low–density lipoprotein cholesterol; MET, metabolic equivalent; TDI, Townsend deprivation index.

# Supplemental Table 6. Associations between the degree of joint risk factor control and risk of CHD, MI, and CVD-related mortality among non-cancer participants.

| Degree of joint risk factor control | No. of cases/total | IR, per 1,000 person–years | Unadjusted model HR (95% CI), *P* value | Multivariable adjusted model HR (95% CI), *P* value |
| --- | --- | --- | --- | --- |
| CHD |  |  |  |  |
| Overall | 4814/58228 | 6.88 | – | – |
| ≤3 controlled risk factors | 1106/8458 | 11.36 | Reference (1.00) | Reference (1.00) |
| 4 controlled risk factors | 1477/14901 | 8.37 | 0.74 (0.68–0.79), <0.001 | 0.80 (0.74–0.86), <0.001 |
| 5 controlled risk factors | 1356/17284 | 6.49 | 0.57 (0.53–0.62), <0.001 | 0.71 (0.65–0.76), <0.001 |
| ≥6 controlled risk factors | 875/17585 | 4.04 | 0.35 (0.32–0.39), <0.001 | 0.55 (0.50–0.60), <0.001 |
| Per one additional controlled risk factor | – | – | 0.75 (0.74–0.77), <0.001 | 0.85 (0.83–0.87), <0.001 |
| MI |  |  |  |  |
| Overall | 1322/58228 | 1.84 | – | – |
| ≤3 controlled risk factors | 322/8458 | 3.16 | Reference (1.00) | Reference (1.00) |
| 4 controlled risk factors | 430/14901 | 2.35 | 0.74 (0.64–0.86), <0.001 | 0.81 (0.70–0.93), 0.003 |
| 5 controlled risk factors | 373/17284 | 1.74 | 0.55 (0.47–0.64), <0.001 | 0.67 (0.58–0.78), <0.001 |
| ≥6 controlled risk factors | 197/17585 | 0.89 | 0.28 (0.24–0.34), <0.001 | 0.43 (0.35–0.51), <0.001 |
| Per one additional controlled risk factor |  |  | 0.71 (0.68–0.74), <0.001 | 0.79 (0.76–0.83), <0.001 |
| CVD-related mortality |  |  |  |  |
| Overall | 919/58228 | 1.26 | – | – |
| ≤3 controlled risk factors | 240/8458 | 2.31 | Reference (1.00) | Reference (1.00) |
| 4 controlled risk factors | 293/14901 | 1.59 | 0.68 (0.58–0.81), <0.001 | 0.75 (0.64–0.90), 0.001 |
| 5 controlled risk factors | 233/17284 | 1.08 | 0.46 (0.39–0.55), <0.001 | 0.60 (0.50–0.72), <0.001 |
| ≥6 controlled risk factors | 153/17585 | 0.69 | 0.30 (0.24–0.36), <0.001 | 0.50 (0.41–0.61), <0.001 |
| Per one additional controlled risk factor | – | – | 0.71 (0.67–0.74), <0.001 | 0.81 (0.77–0.86), <0.001 |

Multivariable model was adjusted for age, sex, ethnicity (White, South Asian, Black, and other), TDI (≥ median, < median, missing), drinking status (daily,1–4 times/weekly, never/rarely, missing), household income (>£52,000, £18,000–52000, <£18,000, and missing), college education (yes/no, missing), family history of cancer (yes/no), family history of cardiovascular disease (yes/no). Abbreviations: IR, incidence rate; HR, hazard ratio; CI, confidence interval.

# Supplemental Table 7. Association between cancer types and risk of incident CHD, MI, and CVD–related mortality.

| Cancer types | No. of cases/total | IR, per 1,000 person–years | HR (95% CI) , *P* value |
| --- | --- | --- | --- |
| CHD |  |  |  |
| Non–cancer | 4814/58228 | 6.88 | Reference (1.00) |
| Hematologic cancer | 133/982 | 13.09 | 1.94 (1.61–2.33), <0.001 |
| Respiratory cancer | 34/240 | 15.01 | 1.66 (1.18–2.33), 0.003 |
| Breast cancer | 236/4092 | 4.99 | 1.19 (1.03–1.37), 0.015 |
| Genital urinary cancer | 367/3300 | 10.01 | 1.05 (0.94–1.18), 0.407 |
| Digestive cancer | 154/1521 | 9.76 | 1.10 (0.92–1.32), 0.279 |
| Skin cancer | 319/3580 | 7.56 | 1.03 (0.92–1.15), 0.610 |
| Other cancer | 55/842 | 6.00 | 1.04 (0.79–1.36), 0.794 |
| MI |  |  |  |
| Non–cancer | 1322/58228 | 1.84 | Reference (1.00) |
| Hematologic cancer | 48/982 | 4.54 | 2.56 (1.87–3.49), <0.001 |
| Respiratory cancer | 10/240 | 4.15 | 1.76 (0.94–3.29), 0.076 |
| Breast cancer | 55/4092 | 1.14 | 1.13 (0.85–1.50), 0.411 |
| Genital urinary cancer | 86/3300 | 2.25 | 0.92 (0.73–1.16), 0.500 |
| Digestive cancer | 50/1521 | 3.05 | 1.40 (1.02–1.91), 0.039 |
| Skin cancer | 75/3580 | 1.72 | 0.87 (0.69–1.10), 0.255 |
| Other cancer | 24/842 | 2.58 | 1.64 (1.09–2.47), 0.017 |
| CVD-related mortality |  |  |  |
| Non–cancer | 919/58228 | 1.26 | Reference (1.00) |
| Hematologic cancer | 19/982 | 1.76 | 1.32 (0.81–2.13), 0.264 |
| Respiratory cancer | 12/240 | 4.94 | 2.64 (1.49–4.69), <0.001 |
| Breast cancer | 27/4092 | 0.56 | 0.77 (0.51–1.15), 0.198 |
| Genital urinary cancer | 59/3300 | 1.52 | 0.78 (0.58–1.03), 0.084 |
| Digestive cancer | 29/1521 | 1.74 | 0.90 (0.59–1.37), 0.625 |
| Skin cancer | 56/3580 | 1.27 | 0.93 (0.71–1.22), 0.612 |
| Other cancer | 9/842 | 0.95 | 0.87 (0.45–1.68), 0.673 |

Model was adjusted for age, sex, ethnicity (White, South Asian, Black, and other), TDI (≥ median, < median, missing), smoking status (current, previous, never), drinking status (daily,1–4 times/weekly, never/rarely, missing), BMI (continuous), household income (>£52,000, £18,000–52000, <£18,000, and missing), college education (yes/no, missing), sleep quality score (3–5 scores, <3 scores, missing), chemotherapy (yes/no), radiotherapy (yes/no), family history of cancer (yes/no), family history of cardiovascular disease (yes/no). Abbreviations: IR, incidence rate; HR, hazard ratio; CI, confidence interval.

# Supplemental Table 8. Subgroup analyses: associations between degree of joint risk factor control and risk of incident CHD among cancer survivors.

|  | HR (95% CI) | | | |  |
| --- | --- | --- | --- | --- | --- |
| Subgroups | ≤3 risk factors | 4 risk factors | 5 risk factors | ≥6 risk factors | P for interaction |
| Sex |  |  |  |  | 0.313 |
| Male | Reference (1.00) | 0.89 (0.75–1.07), 0.224 | 0.75 (0.62–0.91), 0.004 | 0.52 (0.41–0.65), <0.001 |  |
| Female | Reference (1.00) | 0.81 (0.62–1.06), 0.132 | 0.78 (0.60–1.02), 0.073 | 0.71 (0.54–0.95), 0.022 |  |
| Age |  |  |  |  | 0.020 |
| <65years | Reference (1.00) | 0.71 (0.58–0.88), 0.002 | 0.69 (0.56–0.85), <0.001 | 0.49 (0.38–0.61), <0.001 |  |
| ≥65years | Reference (1.00) | 1.06 (0.85–1.31), 0.600 | 0.85 (0.67–1.07), 0.158 | 0.70 (0.54–0.92), 0.010 |  |
| Ethnicity |  |  |  |  | 0.596 |
| White | Reference (1.00) | 0.87 (0.75–1.02), 0.079 | 0.76 (0.65–0.89), <0.001 | 0.60 (0.50–0.71), <0.001 |  |
| Others | Reference (1.00) | 0.79 (0.29–2.16), 0.647 | 0.81 (0.30–2.19), 0.679 | 0.41 (0.12–1.43), 0.164 |  |
| Townsend deprivation index | |  |  |  | 0.384 |
| High | Reference (1.00) | 0.80 (0.65–0.99), 0.039 | 0.79 (0.64–0.98), 0.031 | 0.59 (0.46–0.76), <0.001 |  |
| Low | Reference (1.00) | 0.95 (0.77–1.18), 0.655 | 0.75 (0.59–0.93), 0.011 | 0.60 (0.46–0.78), <0.001 |  |
| Chemotherapy/Radiotherapy | |  |  |  | 0.873 |
| Yes | Reference (1.00) | 0.81 (0.61–1.06), 0.127 | 0.78 (0.59–1.03), 0.083 | 0.58 (0.41–0.80), 0.001 |  |
| No | Reference (1.00) | 0.90 (0.75–1.07), 0.233 | 0.76 (0.63–0.92), 0.004 | 0.58 (0.47–0.72), <0.001 |  |
| Cancer type |  |  |  |  | 0.593 |
| Hematologic cancer | Reference (1.00) | 0.93 (0.56–1.57), 0.798 | 0.96 (0.57–1.61), 0.868 | 0.62 (0.34–1.11), 0.106 |  |
| Respiratory cancer | Reference (1.00) | 0.37 (0.15–0.95), 0.039 | 0.41 (0.14–1.22), 0.108 | 0.44 (0.13–1.45), 0.177 |  |
| Breast cancer | Reference (1.00) | 0.63 (0.43–0.93), 0.019 | 0.81 (0.57–1.16), 0.256 | 0.64 (0.43–0.97), 0.034 |  |
| Genital urinary cancer | Reference (1.00) | 0.88 (0.67–1.15), 0.357 | 0.73 (0.55–0.97), 0.032 | 0.55 (0.39–0.78), <0.001 |  |
| Digestive cancer | Reference (1.00) | 0.82 (0.55–1.24), 0.348 | 0.54 (0.34–0.85), 0.008 | 0.53 (0.32–0.88), 0.015 |  |
| Skin cancer | Reference (1.00) | 1.04 (0.76–1.43), 0.793 | 0.83 (0.60–1.16), 0.277 | 0.59 (0.40–0.86), 0.006 |  |
| Other cancer | Reference (1.00) | 0.93 (0.41–2.11), 0.864 | 0.81 (0.36–1.80), 0.601 | 0.60 (0.25–1.42), 0.241 |  |

The multivariate model adjusted for age, sex, ethnicity (White, South Asian, Black, and other), TDI (≥ median, < median, missing), drinking status (daily,1–4 times/weekly, never/rarely, missing), household income (>£52,000, £18,000–52000, <£18,000, and missing), college education (yes/no, missing), sleep quality score (3–5 scores, <3 scores, missing), chemotherapy (yes/no), radiotherapy (yes/no), cancer type, family history of cancer (yes/no), family history of cardiovascular disease (yes/no), except the grouping variable itself. Abbreviations: HR, hazard ratio; CI, confidence interval.

# Supplemental Table 9. Subgroup analyses: associations between degree of joint risk factor control and risk of incident myocardial infarction among cancer survivors.

|  | HR (95% CI) | | | |  |
| --- | --- | --- | --- | --- | --- |
| Subgroups | ≤3 risk factors | 4 risk factors | 5 risk factors | ≥6 risk factors | P for interaction |
| Sex |  |  |  |  | 0.370 |
| Male | Reference (1.00) | 0.82 (0.59–1.14), 0.228 | 0.69 (0.49–0.98), 0.037 | 0.43 (0.28–0.67), <0.001 |  |
| Female | Reference (1.00) | 0.49 (0.28–0.85), 0.012 | 0.66 (0.40–1.10), 0.112 | 0.39 (0.22–0.71), 0.002 |  |
| Age |  |  |  |  | 0.168 |
| <65years | Reference (1.00) | 0.53 (0.36–0.78), 0.001 | 0.61 (0.42–0.88), 0.008 | 0.34 (0.21–0.53), <0.001 |  |
| ≥65years | Reference (1.00) | 0.97 (0.63–1.47), 0.869 | 0.78 (0.50–1.23), 0.281 | 0.56 (0.32–0.99), 0.046 |  |
| Ethnicity |  |  |  |  | 0.402 |
| White | Reference (1.00) | 0.73 (0.55–0.98), 0.036 | 0.71 (0.53–0.94), 0.019 | 0.43 (0.30–0.61), <0.001 |  |
| Others | Reference (1.00) | 0.11 (0.00–4.56), 0.241 | 0.01 (0.00–0.54), 0.022 | 0.08 (0.00–2.04), 0.127 |  |
| Townsend deprivation index | |  |  |  | 0.936 |
| High | Reference (1.00) | 0.68 (0.45–1.02), 0.060 | 0.63 (0.42–0.96), 0.030 | 0.39 (0.24–0.66), <0.001 |  |
| Low | Reference (1.00) | 0.77 (0.52–1.15), 0.206 | 0.78 (0.52–1.16), 0.222 | 0.46 (0.28–0.76), 0.002 |  |
| Chemotherapy/Radiotherapy | |  |  |  | 0.425 |
| Yes | Reference (1.00) | 0.52 (0.31–0.88), 0.015 | 0.63 (0.38–1.06), 0.081 | 0.30 (0.15–0.59), <0.001 |  |
| No | Reference (1.00) | 0.80 (0.57–1.13), 0.214 | 0.73 (0.52–1.04), 0.080 | 0.47 (0.31–0.71), <0.001 |  |
| Cancer type |  |  |  |  | 0.169 |
| Hematologic cancer | Reference (1.00) | 0.80 (0.37–1.71), 0.567 | 0.56 (0.25–1.27), 0.164 | 0.24 (0.08–0.70), 0.009 |  |
| Respiratory cancer | Reference (1.00) | 0.09 (0.01–1.31), 0.079 | 0.65 (0.09–4.72), 0.672 | 0.57 (0.06–5.28), 0.617 |  |
| Breast cancer | Reference (1.00) | 0.29 (0.12–0.68), 0.004 | 0.68 (0.36–1.30), 0.246 | 0.23 (0.09–0.58), 0.002 |  |
| Genital urinary cancer | Reference (1.00) | 0.72 (0.42–1.25), 0.244 | 0.72 (0.41–1.27), 0.260 | 0.42 (0.20–0.89), 0.023 |  |
| Digestive cancer | Reference (1.00) | 0.46 (0.22–0.96), 0.039 | 0.38 (0.17–0.84), 0.017 | 0.48 (0.21–1.09), 0.079 |  |
| Skin cancer | Reference (1.00) | 1.77 (0.85–3.70), 0.128 | 1.28 (0.60–2.76), 0.522 | 0.77 (0.32–1.88), 0.567 |  |
| Other cancer | Reference (1.00) | 0.89 (0.24–3.27), 0.862 | 0.81 (0.23–2.87), 0.740 | 0.80 (0.21–3.00), 0.744 |  |

The multivariate model adjusted for age, sex, ethnicity (White, South Asian, Black, and other), TDI (≥ median, < median, missing), drinking status (daily,1–4 times/weekly, never/rarely, missing), household income (>£52,000, £18,000–52000, <£18,000, and missing), college education (yes/no, missing), sleep quality score (3–5 scores, <3 scores, missing), chemotherapy (yes/no), radiotherapy (yes/no), cancer type, family history of cancer (yes/no), family history of cardiovascular disease (yes/no), except the grouping variable itself. Abbreviations: HR, hazard ratio; CI, confidence interval.

# Supplemental Table 10. Subgroup analyses: associations between degree of joint risk factor control and risk of incident CVD-related mortality among cancer survivors.

|  | HR (95% CI) | | | |  |
| --- | --- | --- | --- | --- | --- |
| Subgroups | ≤3 risk factors | 4 risk factors | 5 risk factors | ≥6 risk factors | P for interaction |
| Sex |  |  |  |  | 0.811 |
| Male | Reference (1.00) | 0.72 (0.48–1.09), 0.122 | 0.60 (0.38–0.94), 0.027 | 0.48 (0.28–0.84), 0.010 |  |
| Female | Reference (1.00) | 0.65 (0.35–1.22), 0.178 | 0.42 (0.21–0.84), 0.013 | 0.27 (0.12–0.61), 0.001 |  |
| Age |  |  |  |  | 0.245 |
| <65years | Reference (1.00) | 0.59 (0.36–0.97), 0.037 | 0.43 (0.25–0.73), 0.002 | 0.24 (0.12–0.46), <0.001 |  |
| ≥65years | Reference (1.00) | 0.86 (0.53–1.39), 0.526 | 0.65 (0.38–1.12), 0.120 | 0.65 (0.35–1.22), 0.180 |  |
| Townsend deprivation index | |  |  |  | 0.325 |
| High | Reference (1.00) | 0.61 (0.38–0.97), 0.035 | 0.48 (0.29–0.80), 0.005 | 0.48 (0.27–0.85), 0.011 |  |
| Low | Reference (1.00) | 0.88 (0.52–1.48), 0.626 | 0.62 (0.35–1.09), 0.098 | 0.31 (0.14–0.67), 0.003 |  |
| Chemotherapy/Radiotherapy | |  |  |  | 0.010 |
| Yes | Reference (1.00) | 1.09 (0.54–2.18), 0.813 | 1.04 (0.51–2.12), 0.910 | 0.92 (0.42–2.00), 0.827 |  |
| No | Reference (1.00) | 0.61 (0.41–0.92), 0.017 | 0.41 (0.26–0.65), <0.001 | 0.26 (0.14–0.49), <0.001 |  |
| Cancer type |  |  |  |  | 0.636 |
| Hematologic cancer | Reference (1.00) | 0.89 (0.27–2.88), 0.841 | 0.36 (0.08–1.64), 0.188 | 0.41 (0.09–1.90), 0.255 |  |
| Respiratory cancer | Reference (1.00) | 0.14 (0.01–1.33), 0.087 | – | 0.24 (0.02–3.01), 0.269 |  |
| Breast cancer | Reference (1.00) | 0.86 (0.27–2.73), 0.797 | 1.02 (0.34–3.09), 0.976 | 0.78 (0.23–2.68), 0.699 |  |
| Genital urinary cancer | Reference (1.00) | 0.48 (0.25–0.91), 0.025 | 0.43 (0.22–0.85), 0.014 | 0.30 (0.12–0.74), 0.009 |  |
| Digestive cancer | Reference (1.00) | 0.72 (0.29–1.81), 0.488 | 0.64 (0.23–1.72), 0.372 | 0.20 (0.04–0.98), 0.048 |  |
| Skin cancer | Reference (1.00) | 1.13 (0.55–2.35), 0.737 | 0.82 (0.37–1.82), 0.629 | 0.52 (0.20–1.37), 0.187 |  |
| Other cancer | Reference (1.00) | 0.59 (0.06–5.85), 0.656 | 0.77 (0.08–7.11), 0.817 | 0.55 (0.06–5.31), 0.603 |  |

The multivariate model adjusted for age, sex, ethnicity (White, South Asian, Black, and other), TDI (≥ median, < median, missing), drinking status (daily,1–4 times/weekly, never/rarely, missing), household income (>£52,000, £18,000–52000, <£18,000, and missing), college education (yes/no, missing), sleep quality score (3–5 scores, <3 scores, missing), chemotherapy (yes/no), radiotherapy (yes/no), cancer type, family history of cancer (yes/no), family history of cardiovascular disease (yes/no), except the grouping variable itself. Abbreviations: HR, hazard ratio; CI, confidence interval.

# Supplemental Table 11. Sensitivity analyses: associations between degree of joint risk factor control and risk of CHD, MI, and CVD-related mortality among cancer survivors after excluding events occurring within the first 5 years of follow–up (n=14,026).

| Degree of joint risk factor control | No. of cases/total | IR, per 1,000 person–years | Unadjusted model HR (95% CI), *P* value | Multivariable adjusted model HR (95% CI), *P* value |
| --- | --- | --- | --- | --- |
| CHD |  |  |  |  |
| ≤3 risk factors | 189/2115 | 8.06 | Reference (1.00) | Reference (1.00) |
| 4 risk factors | 225/3573 | 5.47 | 0.67 (0.55–0.81), <0.001 | 0.75 (0.62–0.91), 0.004 |
| 5 risk factors | 232/4181 | 4.79 | 0.58 (0.48–0.70), <0.001 | 0.74 (0.61–0.91), 0.003 |
| ≥6 risk factors | 149/4157 | 3.03 | 0.36 (0.29–0.45), <0.001 | 0.59 (0.47–0.74), <0.001 |
| Per one additional controlled risk factor | 795/14026 | 4.90 | 0.77 (0.73–0.82), <0.001 | 0.88 (0.83–0.94), <0.001 |
| MI |  |  |  |  |
| ≤3 risk factors | 61/2115 | 2.55 | Reference (1.00) | Reference (1.00) |
| 4 risk factors | 57/3573 | 1.36 | 0.53 (0.37–0.76), <0.001 | 0.56 (0.39–0.80), 0.002 |
| 5 risk factors | 66/4181 | 1.35 | 0.52 (0.37–0.73), <0.001 | 0.60 (0.42–0.86), 0.005 |
| ≥6 risk factors | 29/4157 | 0.58 | 0.22 (0.14–0.35), <0.001 | 0.31 (0.20–0.49), <0.001 |
| Per one additional controlled risk factor | 213/14026 | 1.30 | 0.69 (0.62–0.77), <0.001 | 0.75 (0.67–0.84), <0.001 |
| CVD-related mortality |  |  |  |  |
| ≤3 risk factors | 46/2115 | 1.91 | Reference (1.00) | Reference (1.00) |
| 4 risk factors | 52/3573 | 1.24 | 0.64 (0.43–0.95), 0.028 | 0.74 (0.49–1.10), 0.134 |
| 5 risk factors | 39/4181 | 0.79 | 0.41 (0.27–0.63), <0.001 | 0.55 (0.36–0.86), 0.008 |
| ≥6 risk factors | 24/4157 | 0.48 | 0.25 (0.15–0.40), <0.001 | 0.43 (0.26–0.72), 0.001 |
| Per one additional controlled risk factor | 161/14026 | 0.98 | 0.67 (0.59–0.75), <0.001 | 0.78 (0.68–0.89), <0.001 |

Multivariable model was adjusted for age, sex, ethnicity (White, South Asian, Black, and other), TDI (≥ median, < median, missing), drinking status (daily,1–4 times/weekly, never/rarely, missing), household income (>£52,000, £18,000–52000, <£18,000, and missing), college education (yes/no, missing), chemotherapy (yes/no), radiotherapy (yes/no), cancer type (hematologic cancer, respiratory cancer, breast cancer, genital urinary cancer, digestive cancer, skin cancer, and other cancer), family history of cancer (yes/no), family history of cardiovascular disease (yes/no).

Abbreviations: IR, incidence rate; HR, hazard ratio; CI, confidence interval.

# Supplemental Table 12. Sensitivity analyses: associations between degree of joint risk factor control and risk of CHD, MI, and CVD-related mortality among cancer survivors using Fine & Gray Models for competing risk.

| Degree of joint risk factor control | No. of cases/total | IR, per 1,000 person–years | Unadjusted model HR (95% CI), *P* value | Multivariable adjusted model HR (95% CI), *P* value |
| --- | --- | --- | --- | --- |
| CHD |  |  |  |  |
| ≤3 risk factors | 307/2242 | 12.93 | Reference (1.00) | Reference (1.00) |
| 4 risk factors | 407/3764 | 9.77 | 0.75 (0.65–0.87), <0.001 | 0.87 (0.75–1.01), 0.072 |
| 5 risk factors | 363/4320 | 7.43 | 0.57 (0.49–0.67), <0.001 | 0.76 (0.65–0.89), <0.001 |
| ≥6 risk factors | 221/4231 | 4.48 | 0.35 (0.29–0.41), <0.001 | 0.59 (0.49–0.71), <0.001 |
| Per one additional controlled risk factor | 1298/14557 | 7.93 | 0.75 (0.72–0.79), <0.001 | 0.87 (0.83–0.91), <0.001 |
| MI |  |  |  |  |
| ≤3 risk factors | 91/2242 | 3.64 | Reference (1.00) | Reference (1.00) |
| 4 risk factors | 103/3764 | 2.37 | 0.65 (0.49–0.86), 0.003 | 0.71 (0.53–0.94), 0.018 |
| 5 risk factors | 103/4320 | 2.05 | 0.56 (0.42–0.74), <0.001 | 0.69 (0.51–0.92), 0.011 |
| ≥6 risk factors | 51/4231 | 1.02 | 0.28 (0.20–0.39), <0.001 | 0.42 (0.29–0.59), <0.001 |
| Per one additional controlled risk factor | 348/14557 | 2.06 | 0.73 (0.67–0.79), <0.001 | 0.81 (0.75–0.89), <0.001 |
| CVD-related mortality |  |  |  |  |
| ≤3 risk factors | 64/2242 | 2.52 | Reference (1.00) | Reference (1.00) |
| 4 risk factors | 69/3764 | 1.57 | 0.62 (0.44–0.87), 0.005 | 0.72 (0.51–1.00), 0.053 |
| 5 risk factors | 50/4320 | 0.98 | 0.39 (0.27–0.56), <0.001 | 0.54 (0.37–0.79), 0.001 |
| ≥6 risk factors | 28/4231 | 0.55 | 0.22 (0.14–0.34), <0.001 | 0.41 (0.26–0.66), <0.001 |
| Per one additional controlled risk factor | 211/14557 | 1.24 | 0.65 (0.58–0.73), <0.001 | 0.78 (0.69–0.88), <0.001 |

Multivariable model was adjusted for age, sex, ethnicity (White, South Asian, Black, and other), TDI (≥ median, < median, missing), drinking status (daily,1–4 times/weekly, never/rarely, missing), household income (>£52,000, £18,000–52000, <£18,000, and missing), college education (yes/no, missing), chemotherapy (yes/no), radiotherapy (yes/no), cancer type (hematologic cancer, respiratory cancer, breast cancer, genital urinary cancer, digestive cancer, skin cancer, and other cancer), family history of cancer (yes/no), family history of cardiovascular disease (yes/no). Abbreviations: IR, incidence rate; HR, hazard ratio; CI, confidence interval.

# Supplemental Table 13. Multiple-imputation sensitivity analyses of joint risk factor control and risks of CHD, MI, and CVD-related mortality among cancer survivors.

| Degree of joint risk factor control | No. of cases/total | IR, per 1,000 person–years | Multivariable adjusted model HR (95% CI), *P* value |
| --- | --- | --- | --- |
| CHD |  |  |  |
| ≤3 risk factors | 307/2242 | 12.93 | Reference (1.00) |
| 4 risk factors | 407/3764 | 9.77 | 0.87 (0.75–1.01), 0.068 |
| 5 risk factors | 363/4320 | 7.43 | 0.76 (0.66–0.89), <0.001 |
| ≥6 risk factors | 221/4231 | 4.48 | 0.59 (0.49–0.70), <0.001 |
| Per one additional controlled risk factor | 1298/14557 | 7.93 | 0.87 (0.83–0.91), <0.001 |
| MI |  |  |  |
| ≤3 risk factors | 91/2242 | 3.64 | Reference (1.00) |
| 4 risk factors | 103/3764 | 2.37 | 0.71 (0.53–0.94), 0.018 |
| 5 risk factors | 103/4320 | 2.05 | 0.68 (0.51–0.91), 0.010 |
| ≥6 risk factors | 51/4231 | 1.02 | 0.42 (0.29–0.59), <0.001 |
| Per one additional controlled risk factor | 348/14557 | 2.06 | 0.81 (0.74–0.89), <0.001 |
| CVD-related mortality |  |  |  |
| ≤3 risk factors | 64/2242 | 2.52 | Reference (1.00) |
| 4 risk factors | 69/3764 | 1.57 | 0.72 (0.51–1.01), 0.059 |
| 5 risk factors | 50/4320 | 0.98 | 0.55 (0.37–0.80), 0.002 |
| ≥6 risk factors | 28/4231 | 0.55 | 0.41 (0.26–0.65), <0.001 |
| Per one additional controlled risk factor | 211/14557 | 1.24 | 0.78 (0.69–0.87), <0.001 |

Multivariable model was adjusted for age, sex, ethnicity (White, South Asian, Black, and other), TDI (≥ median, < median, missing), drinking status (daily,1–4 times/weekly, never/rarely, missing), household income (>£52,000, £18,000–52000, <£18,000, and missing), college education (yes/no, missing), chemotherapy (yes/no), radiotherapy (yes/no), cancer type (hematologic cancer, respiratory cancer, breast cancer, genital urinary cancer, digestive cancer, skin cancer, and other cancer), family history of cancer (yes/no), family history of cardiovascular disease (yes/no). Abbreviations: IR, incidence rate; HR, hazard ratio; CI, confidence interval.

# Supplemental Table 14. Sensitivity analyses: associations between degree of joint risk factor control and risk of CHD, MI, and CVD-related mortality compared to matched control group after excluding events occurring within the first 5 years of follow–up (n=70,348).

| Degree of joint risk factor control | No. of cases/total | IR, per 1,000 person–years | Unadjusted model HR (95% CI), *P* value | Multivariable adjusted model HR (95% CI), *P* value |
| --- | --- | --- | --- | --- |
| CHD |  |  |  |  |
| Non–cancer | 3085/56322 | 4.44 | Reference (1.00) | Reference (1.00) |
| ≤3 risk factors | 189/2115 | 8.06 | 1.90 (1.64–2.20), <0.001 | 1.46 (1.26–1.69), <0.001 |
| 4 risk factors | 225/3573 | 5.47 | 1.27 (1.11–1.45), <0.001 | 1.11 (0.97–1.28), 0.12 |
| 5 risk factors | 232/4181 | 4.79 | 1.10 (0.97–1.26), 0.151 | 1.12 (0.98–1.28), 0.088 |
| ≥6 risk factors | 149/4157 | 3.03 | 0.69 (0.59–0.82), <0.001 | 0.91 (0.77–1.07), 0.262 |
| MI |  |  |  |  |
| Non–cancer | 845/56322 | 1.20 | Reference (1.00) | Reference (1.00) |
| ≤3 risk factors | 61/2115 | 2.55 | 2.22 (1.71–2.87), <0.001 | 1.75 (1.35–2.28), <0.001 |
| 4 risk factors | 57/3573 | 1.36 | 1.17 (0.89–1.53), 0.259 | 1.03 (0.79–1.35), 0.802 |
| 5 risk factors | 66/4181 | 1.35 | 1.15 (0.89–1.47), 0.280 | 1.16 (0.90–1.49), 0.244 |
| ≥6 risk factors | 29/4157 | 0.58 | 0.49 (0.34–0.72), <0.001 | 0.62 (0.43–0.90), 0.012 |
| CVD-related mortality |  |  |  |  |
| Non–cancer | 630/56322 | 0.89 | Reference (1.00) | Reference (1.00) |
| ≤3 risk factors | 46/2115 | 1.91 | 2.24 (1.66–3.02), <0.001 | 1.63 (1.21–2.20), 0.001 |
| 4 risk factors | 52/3573 | 1.24 | 1.43 (1.08–1.90), 0.013 | 1.22 (0.92–1.61), 0.176 |
| 5 risk factors | 39/4181 | 0.79 | 0.91 (0.66–1.26), 0.582 | 0.93 (0.67–1.28), 0.659 |
| ≥6 risk factors | 24/4157 | 0.48 | 0.55 (0.37–0.83), 0.004 | 0.76 (0.51–1.14), 0.188 |

Multivariable model was adjusted for age, sex, ethnicity (White, South Asian, Black, and other), TDI (≥ median, < median, missing), drinking status (daily,1–4 times/weekly, never/rarely, missing), household income (>£52,000, £18,000–52000, <£18,000, and missing), college education (yes/no, missing), family history of cancer (yes/no), family history of cardiovascular disease (yes/no).

Abbreviations: IR, incidence rate; HR, hazard ratio; CI, confidence interval.

# Supplemental Table 15. Sensitivity analyses: associations between degree of joint risk factor control and risk of coronary heart disease compared to matched control group using Fine & Gray Models for competing risk.

| Degree of joint risk factor control | No. of cases/total | IR, per 1,000 person–years | Unadjusted model HR (95% CI), *P* value | Multivariable adjusted model HR (95% CI), *P* value |
| --- | --- | --- | --- | --- |
| CHD |  |  |  |  |
| Non–cancer | 4814/58228 | 6.88 | Reference (1.00) | Reference (1.00) |
| ≤3 risk factors | 307/2242 | 12.93 | 1.89 (1.69–2.12), <0.001 | 1.43 (1.27–1.61), <0.001 |
| 4 risk factors | 407/3764 | 9.77 | 1.43 (1.29–1.58), <0.001 | 1.25 (1.12–1.38), <0.001 |
| 5 risk factors | 363/4320 | 7.43 | 1.08 (0.97–1.20), 0.144 | 1.10 (0.99–1.23), 0.072 |
| ≥6 risk factors | 221/4231 | 4.48 | 0.65 (0.57–0.75), <0.001 | 0.87 (0.76–1.00), 0.043 |
| MI |  |  |  |  |
| Non–cancer | 1322/58228 | 1.84 | Reference (1.00) | Reference (1.00) |
| ≤3 risk factors | 91/2242 | 3.64 | 2.01 (1.63–2.49), <0.001 | 1.56 (1.26–1.94), <0.001 |
| 4 risk factors | 103/3764 | 2.37 | 1.30 (1.07–1.59), 0.010 | 1.14 (0.93–1.39), 0.211 |
| 5 risk factors | 103/4320 | 2.05 | 1.13 (0.92–1.38), 0.247 | 1.13 (0.93–1.39), 0.221 |
| ≥6 risk factors | 51/4231 | 1.02 | 0.56 (0.42–0.74), <0.001 | 0.71 (0.54–0.95), 0.019 |
| CVD-related mortality |  |  |  |  |
| Non–cancer | 919/58228 | 1.26 | Reference (1.00) | Reference (1.00) |
| ≤3 risk factors | 64/2242 | 2.52 | 2.04 (1.58–2.63), <0.001 | 1.46 (1.13–1.88), 0.004 |
| 4 risk factors | 69/3764 | 1.57 | 1.26 (0.99–1.61), 0.064 | 1.06 (0.83–1.35), 0.666 |
| 5 risk factors | 50/4320 | 0.98 | 0.79 (0.59–1.05), 0.105 | 0.80 (0.60–1.07), 0.134 |
| ≥6 risk factors | 28/4231 | 0.55 | 0.44 (0.30–0.64), <0.001 | 0.62 (0.42–0.90), 0.012 |

Multivariable model was adjusted for age, sex, ethnicity (White, South Asian, Black, and other), TDI (≥ median, < median, missing), drinking status (daily,1–4 times/weekly, never/rarely, missing), household income (>£52,000, £18,000–52000, <£18,000, and missing), college education (yes/no, missing), family history of cancer (yes/no), family history of cardiovascular disease (yes/no).

Abbreviations: IR, incidence rate; HR, hazard ratio; CI, confidence interval.

# Supplemental Table 16. Multiple-imputation sensitivity analyses of the associations between joint risk factor control and risks of CHD, MI, and CVD-related mortality among cancer survivors compared with matched non-cancer controls.

| Degree of joint risk factor control | No. of cases/total | IR, per 1,000 person–years | Unadjusted model HR (95% CI), *P* value | Multivariable adjusted model HR (95% CI), *P* value |
| --- | --- | --- | --- | --- |
| CHD |  |  |  |  |
| Non–cancer | 4814/58228 | 6.88 | Reference (1.00) | Reference (1.00) |
| ≤3 risk factors | 307/2242 | 12.93 | 1.89 (1.69–2.12), <0.001 | 1.43 (1.27–1.60), <0.001 |
| 4 risk factors | 407/3764 | 9.77 | 1.43 (1.29–1.58), <0.001 | 1.25 (1.13–1.38), <0.001 |
| 5 risk factors | 363/4320 | 7.43 | 1.08 (0.97–1.20), 0.144 | 1.11 (0.99–1.23), 0.066 |
| ≥6 risk factors | 221/4231 | 4.48 | 0.65 (0.57–0.75), <0.001 | 0.87 (0.76–0.99), 0.041 |
| MI |  |  |  |  |
| Non–cancer | 1322/58228 | 1.84 | Reference (1.00) | Reference (1.00) |
| ≤3 risk factors | 91/2242 | 3.64 | 2.01 (1.62–2.48), <0.001 | 1.56 (1.26–1.94), <0.001 |
| 4 risk factors | 103/3764 | 2.37 | 1.30 (1.06–1.59), 0.010 | 1.14 (0.93–1.39), 0.209 |
| 5 risk factors | 103/4320 | 2.05 | 1.12 (0.92–1.37), 0.259 | 1.13 (0.93–1.38), 0.229 |
| ≥6 risk factors | 51/4231 | 1.02 | 0.56 (0.42–0.73), <0.001 | 0.71 (0.54–0.94), 0.017 |
| CVD-related mortality |  |  |  |  |
| Non–cancer | 919/58228 | 1.26 | Reference (1.00) | Reference (1.00) |
| ≤3 risk factors | 64/2242 | 2.52 | 2.04 (1.58–2.63), <0.001 | 1.45 (1.13–1.87), 0.004 |
| 4 risk factors | 69/3764 | 1.57 | 1.26 (0.99–1.61), 0.064 | 1.05 (0.82–1.35), 0.681 |
| 5 risk factors | 50/4320 | 0.98 | 0.79 (0.59–1.05), 0.105 | 0.80 (0.60–1.07), 0.130 |
| ≥6 risk factors | 28/4231 | 0.55 | 0.44 (0.30–0.64), <0.001 | 0.62 (0.42–0.90), 0.013 |

Multivariable model was adjusted for age, sex, ethnicity (White, South Asian, Black, and other), TDI (≥ median, < median, missing), drinking status (daily,1–4 times/weekly, never/rarely, missing), household income (>£52,000, £18,000–52000, <£18,000, and missing), college education (yes/no, missing), family history of cancer (yes/no), family history of cardiovascular disease (yes/no).

Abbreviations: IR, incidence rate; HR, hazard ratio; CI, confidence interval.

# Supplemental Table 17. Sensitivity analyses: associations between degree of joint risk factor control and risk of coronary heart disease following exclusion of initially cancer–free participants who developed cancer during follow-up (n=62,541)

| Degree of joint risk factor control | No. of cases/total | IR, per 1,000 person–years | Unadjusted model HR (95% CI), *P* value | Multivariable adjusted model HR (95% CI), *P* value |
| --- | --- | --- | --- | --- |
| CHD |  |  |  |  |
| Non–cancer | 3621/47984 | 6.18 | Reference (1.00) | Reference (1.00) |
| ≤3 risk factors | 307/2242 | 12.93 | 2.11 (1.88–2.37), <0.001 | 1.50 (1.34–1.69), <0.001 |
| 4 risk factors | 407/3764 | 9.77 | 1.59 (1.43–1.76), <0.001 | 1.32 (1.19–1.46), <0.001 |
| 5 risk factors | 363/4320 | 7.43 | 1.21 (1.08–1.34), <0.001 | 1.17 (1.05–1.30), 0.005 |
| ≥6 risk factors | 221/4231 | 4.48 | 0.73 (0.63–0.83), <0.001 | 0.92 (0.80–1.05), 0.212 |
| MI |  |  |  |  |
| Non–cancer | 1026/47984 | 1.70 | Reference (1.00) | Reference (1.00) |
| ≤3 risk factors | 91/2242 | 3.64 | 2.16 (1.75–2.68), <0.001 | 1.60 (1.29–1.99), <0.001 |
| 4 risk factors | 103/3764 | 2.37 | 1.40 (1.15–1.72), 0.001 | 1.17 (0.95–1.43), 0.131 |
| 5 risk factors | 103/4320 | 2.05 | 1.21 (0.99–1.48), 0.063 | 1.16 (0.95–1.42), 0.145 |
| ≥6 risk factors | 51/4231 | 1.02 | 0.60 (0.45–0.79), <0.001 | 0.73 (0.55–0.97), 0.030 |
| CVD-related mortality |  |  |  |  |
| Non–cancer | 775/47984 | 1.28 | Reference (1.00) | Reference (1.00) |
| ≤3 risk factors | 64/2242 | 2.52 | 2.03 (1.57–2.61), <0.001 | 1.34 (1.04–1.73), 0.026 |
| 4 risk factors | 69/3764 | 1.57 | 1.25 (0.98–1.60), 0.073 | 0.98 (0.77–1.26), 0.882 |
| 5 risk factors | 50/4320 | 0.98 | 0.79 (0.59–1.05), 0.099 | 0.75 (0.56–1.00), 0.048 |
| ≥6 risk factors | 28/4231 | 0.55 | 0.44 (0.30–0.64), <0.001 | 0.58 (0.40–0.85), 0.005 |

Multivariable model was adjusted for age, sex, ethnicity (White, South Asian, Black, and other), TDI (≥ median, < median, missing), drinking status (daily,1–4 times/weekly, never/rarely, missing), household income (>£52,000, £18,000–52000, <£18,000, and missing), college education (yes/no, missing), family history of cancer (yes/no), family history of cardiovascular disease (yes/no).

Abbreviations: IR, incidence rate; HR, hazard ratio; CI, confidence interval.

# Supplemental Table 18. Sensitivity analysis of residual life expectancy estimates using alternative spline degrees of freedom in Royston–Parmar flexible parametric survival models

| Degrees of freedom | Age at estimation, years | Matched non-cancer controls | Cancer survivors by degree of risk factor control | | | |
| --- | --- | --- | --- | --- | --- | --- |
|  |  |  | ≤3 risk factors controlled | 4 risk factors controlled | 5 risk factors controlled | ≥6 risk factors controlled |
| 3 | 50 | 39.81 | 30.41 | 32.85 | 33.38 | 33.72 |
| 3 | 60 | 30.64 | 22.19 | 24.35 | 24.82 | 25.13 |
| 4 | 50 | 39.93 | 30.47 | 32.94 | 33.47 | 33.83 |
| 4 | 60 | 30.78 | 22.31 | 24.49 | 24.96 | 25.28 |
| 5 | 50 | 40.00 | 30.51 | 32.99 | 33.53 | 33.89 |
| 5 | 60 | 30.87 | 22.38 | 24.57 | 25.04 | 25.36 |

Values represent residual life expectancy (years) estimated using Royston–Parmar flexible parametric survival models. Sensitivity analyses were performed using alternative spline specifications with 3, 4, and 5 degrees of freedom.
